# Approaches for Positioning the Active Medium in Hybrid Nanoplasmonics. Focus on Plasmon-Assisted Photopolymerization

**DOI:** 10.1021/acsphotonics.4c00868

**Published:** 2024-08-22

**Authors:** Minyu Chen, Sylvie Marguet, Ali Issa, Safi Jradi, Christophe Couteau, Céline Fiorini-Debuisschert, Ludovic Douillard, Olivier Soppera, Dandan Ge, Jérôme Plain, Xuan Zhou, Cuong Dang, Jérémie Béal, Sergei Kostcheev, Régis Déturche, Tao Xu, Bin Wei, Renaud Bachelot

**Affiliations:** † School of Mechatronic Engineering and Automation, Key Lab of Advanced Display and System Application, Ministry of Education, Shanghai University, Shanghai 2000072, PR China; ‡ Light, Nanomaterials & Nanotechnologies (L2n) Laboratory, CNRS UMR 7076. University of Technology of Troyes-UTT, 12 rue Marie Curie, Troyes Cedex F-10004, France; § Université Paris Saclay, CEA, CNRS, NIMBE, Gif sur Yvette F-91191, France; ∥ Université Paris Saclay, CEA, CNRS, SPEC, Gif sur Yvette F-91191, France; ⊥ Université de Haute Alsace, CNRS, IS2M UMR 7361, Mulhouse F-68100, France; # Université de Strasbourg, Strasbourg cedex F-67081, France; ∇ CNRS-International-NTU-Thales Research Alliance (CINTRA), IRL 3288, 50 Nanyang Drive, Singapore 637553, Singapore; ○ School of Electrical and Electronic Engineering, Nanyang Technological University, Nanyang Avenue, Singapore 639798, Singapore; ◆ Sino-European School of Technology, Shanghai University, Shanghai 200444, PR China

**Keywords:** photopolymerization, plasmon, active medium, hybrid nanoplasmonics, nanophotonics

## Abstract

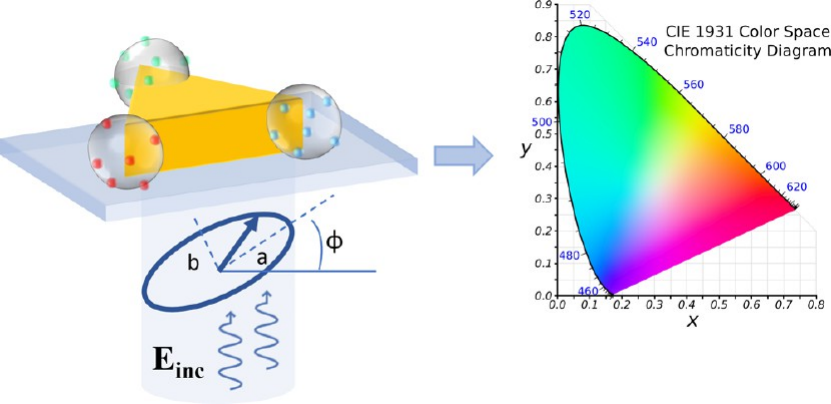

Over the past 20 years, hybrid plasmonics for nanoemitters of light or for nanoabsorbers, based on weak or strong coupling between metallic nanocavities and active media (emissive or absorbing entities), have given rise to important research efforts. One of the main current challenges is the control of the nanoscale spatial distribution and associated symmetry of the active medium in the vicinity of the metallic nanoparticles. In this review, we first recall the main principles of weak and strong coupling by stressing the importance of controlling the spatial distribution of the active medium and present the main approaches developed for achieving this control. Nine different approaches are identified. We then focus our attention on one of them based on plasmonic photopolymerization and discuss the flexibility of this approach in terms of control of the spatial symmetry of the hybrid nanosystem metal–polymer nanoemitters and the resulting polarization dependence of the light emission. The different approaches are analyzed and compared with each other, and some future perspectives and challenges are finally discussed.

## Introduction: The Problematic

The current fast development of nanophotonics^1,2^ requires efficient light emitters or absorbers to be compatible with nanophotonic systems. In particular, nanosources of light are ideally expected to be scalable, tunable, and pretty easy to develop and integrate. Over the past two decades, a promising family of nanosources has emerged and has been giving rise to an increasing interest, namely, Hybrid Plasmonic Nanosystems (HPNs) that are based on the coupling between active quantum nanoemitters/absorbers (such as organic molecules or semiconductor nanocrystals) and metal nanocavities.^3−5^ The latter are generally considered as optical nanoantennas that can couple with molecules or nanocrystals and convert near-field to far-field and vice et versa.^6^

**Figure 1:**
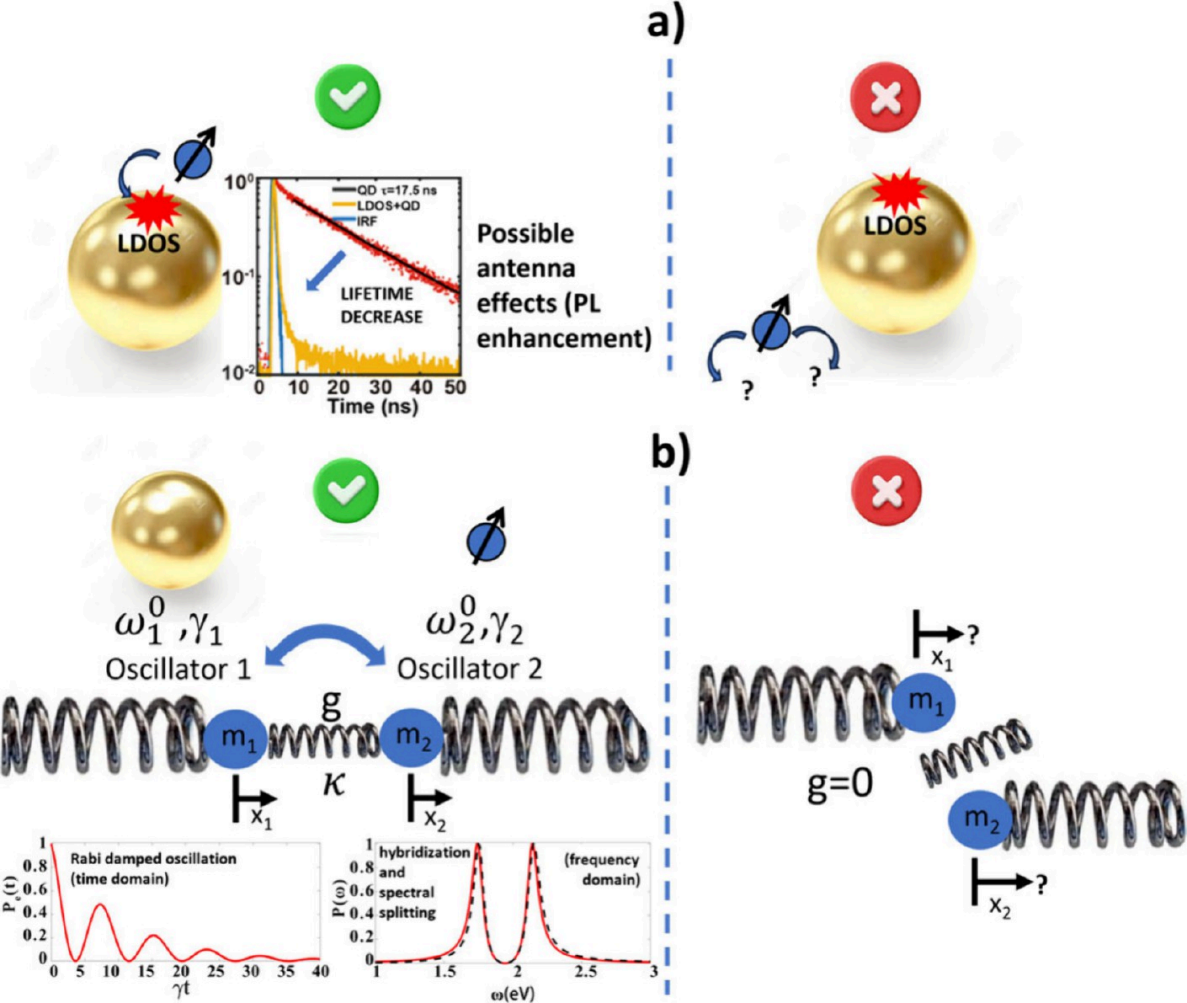
Illustration of the two main kinds of coupling in hybrid plasmonic nanosystems. (a) Weak coupling regime characterized by Purcell and antenna effects. The red region represents the Local Density Of States (LDOS). Left: good spatial alignment between the emitter/absorber and the nanoparticle LDOS. The curve showing a decrease of the fluorescence lifetime with the presence of the LDOS is adapted with permission from ref (3). Copyright 2020, The Author(s), licensed under a CC-BY Creative Commons Attribution 4.0 License. QD: Quantum Dots, IRF: Instrument Response Function. Right: misalignment of the nanosystems. (b) Strong coupling regime represented by two oscillators that are linked together through the coupling strength *g*. This coupling regime is characterized by hybridization of light-matter modes, Rabi oscillation, and spectral splitting. Adapted from ref (7). Copyright 2020 The Author(s), licensed under a CC-BY Creative Commons Attribution 4.0 License. Left: good spatial alignment between both oscillators. Right: misalignment of the two oscillators.

The coupling between quantum nanoemitters (or nanoabsorbers) and metal nanoparticles has been used for controlling light emission and absorption. Two main kinds of coupling, illustrated in Figure 1 and discussed below, were extensively considered, and studied: the weak coupling and the strong coupling.

Regardless of the nature of the coupling and despite the numerous achievements reported so far, an important challenge remains to be addressed: controlling the spatial distribution of the active medium at the nanoscale. Taking up this challenge would allow one, for example, to control the symmetry and the anisotropy of the HPN and to utilize the incident polarization as a rapid and efficient remote optical control of light emission from this hybrid system while minimizing the background emission. One of the main challenges is to control the spatial overlap between the active medium (the quantum nanoemitters or absorbers) and the modes of the plasmonic nanocavity. In microoptoelectronics, this spatial overlap issue is not new. For example, in a distributed-feedback laser (DFB laser), the entire resonator consists of a periodic structure in the laser gain medium, which acts as a distributed Bragg reflector in the wavelength range of the lasing action.^8^ As another example, to efficiently excite a specific mode of an optical fiber, the incident field must spatially overlap, in both amplitude and phase, with the intrinsic mode to be excited.^9^

In plasmonic hybrid nanosystems, controlling this overlap is a difficult task, which is limited by the experimental methods used for integrating the active medium in the vicinity of metal nanoparticles at the nanoscale.

Our review deals with this issue. We first recall the main principles of weak and strong coupling by stressing the importance of controlling the spatial distribution of the active medium. Second, we present the main approaches developed for achieving this control, with nine types of approaches identified. We then focus our attention onto one of them based on plasmonic photopolymerization and discuss the flexibility of this approach in terms of control of the spatial symmetry of the hybrid nanosystem and the resulting polarization dependence of the light emission. We compare the different approaches with each other and develop future challenges and perspectives concerning hybrid plasmonic nanosources of light.

### Case of Weak Coupling (Figure 1a)

**Figure 2:**
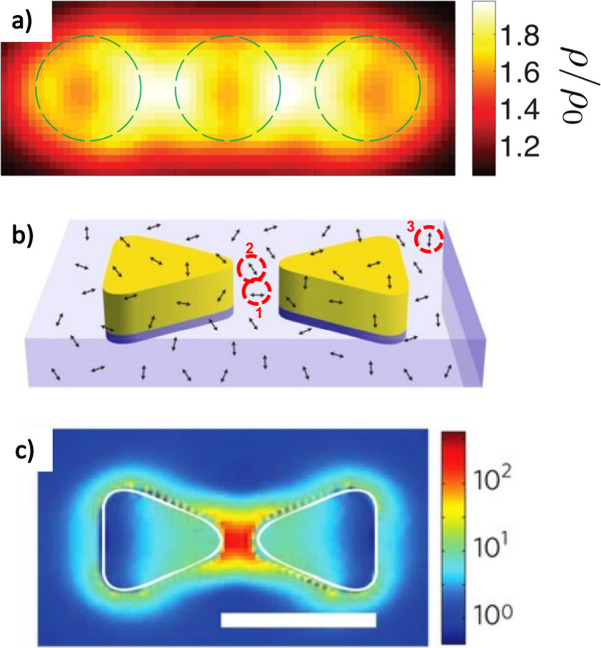
Illustration of the issue of controlling the nanoemitter’s position in the case of weak coupling. (a) Calculated LDOS of a trimer made of gold cylinders (dashed lines) with a diameter of 150 and 30 nm thickness. Adapted with permission from ref (20). Copyright 2013 Optical Society of America. (b) Gold bowtie covered by molecules randomly distributed and randomly oriented. Three molecules, labeled 1, 2, and 3, are highlighted for discussion (see text). (c) Calculated map of the gap mode (field intensity at λ = 780 nm) excited while the bowtie is shined with a polarization parallel to its longitudinal axis. The scale bar represents 100 nm. (b, c) Reproduced with permission from ref (4). Copyright 2009 Springer Nature Limited.

Most of the reported nanoscale couplings achieved in the past are in the weak-coupling regime. Over the past 20 years, this regime has been widely described in many articles including comprehensive review papers.^10−15^ In the case of weak coupling, the Local Density Of States (LDOS, i.e., the electromagnetic states that can be occupied locally by photons) of the metal nanocavity acts as channels of de-excitation for the excited state of the quantum emitter, resulting in an increase in the total de-excitation rate and a decrease in the state lifetime.^16^ This weak coupling regime, illustrated on the left-hand side of Figure 1a, results in a modification of the spontaneous emission rate. It is characterized through the analysis of both fluorescence and lifetime. Fluorescence can be either enhanced or quenched.^4,17,18^ This effect results from the Fermi golden rule (eq 1), initially described by P. A. M. Dirac,^19^ that describes the way an excited emitter de-excites in the presence of a given surrounding LDOS:
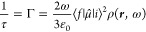
1where τ and Γ are the lifetime and the total de-excitation rate of the quantum emitter, respectively. *i* and *f* stand for the initial excited state and the final lower state, respectively. μ̂ represents the emitter dipole moment related to the perturbation matrix element and the interaction Hamiltonian between both states. ρ is the LDOS at position **r** and frequency ω. Hence, Γ (that includes both radiative and nonradiative relaxation) is proportional to the LDOS. The latter is generally highly spatially dependent as illustrated by Figure 2a, which is an example of a calculated LDOS of a trimer of coupled gold (Au) nanodisks.^20^ In this case, the LDOS is maximum at the gaps, and only emitters located within the gaps are expected to be significantly affected. In the case of a nanocavity presenting a well-defined photonic LDOS, eq 1 can be rewritten for each cavity eigenmode:^16^
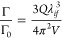
2where Γ_0_ is the free-space emitter de-excitation rate (without the presence of the nanocavity), λ_if_ is the wavelength transition between states *i* and *f*, *Q* is the quality factor of the mode, related to the damping, and *V* is the mode volume. In the case of a plasmonic metal nanocavity, the *Q* factor is generally weak (rarely greater than 70),^21^ but the mode volume can be very small, making the LDOS high, resulting in a strong decrease of τ. This effect is known as the “Purcell effect” because the change in Γ/Γ_0_ due to a structured environment was initially introduced by E. M. Purcell in the case of a single-mode cavity.^22^ The ratio Γ/Γ_0_ is named the “Purcell factor”. This weak coupling effect reminds us that the lifetime of an emitter is not an intrinsic concept, and it rather depends on the emitter’s environment. In Equation 2 the involved mode of volume *V* is generally highly localized, as illustrated in Figure 2b,c that shows the gap mode of a bowtie antenna. This is why the spatial position of the active medium must be controlled too. Note that, in this shown example from ref (4), this mode was used for locally exciting molecules, but it could have been used for the Purcell effect as well, *i.e.*, be used for tailoring the de-excitation of the emitter.

Light emission is affected by the weak coupling, and Equation 3 illustrates the importance of the control of the spatial distribution of the active medium in this context:
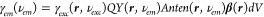
3where γ_em_ is the rate of emission of the HPN at ν_em_ light frequency from an elementary volume *dV* at position **r**, γ_exc_ is the rate of excitation, and ν_exc_ is the frequency of the exciting field absorbed by the emitter, within its absorption band, through one-or-multi photon absorption. γ_exc_ has a spatial dependence because it depends on the local plasmonic near field **E(r**,ν_exc._) at ν_exc_. More specifically, in the case of one-photon absorption, γ_exc_ is proportional to |***μ***·***E***|^2^, where **μ** is the transition dipole of the emitter. QY is the nanoemitter quantum yield and has also a spatial dependence. Indeed, it is equal to Γ_rad_/Γ, where Γ_rad_ is the radiative rate of de-excitation at frequency ν_em_ and Γ (=Γ_rad_ + Γ_nrad_, where Γ_nrad_ is the deexcitation rate of nonradiative processes) is proportional to the LDOS (see eq 1), as discussed previously.

Hence, the quantum yield of the active medium can be modified by its spatial environment at **r**. For example, QY can go to zero if the emitter is too close to the plasmonic cavity: the nonradiative processes dominate and the photoluminescence (PL) gets quenched.^23^ This emitter-metal distance issue is important and will be further discussed. The term *Anten*(***r***, ν_*em*_) represents the antenna effect that the emitted light can experience. The emitted light at ν_*em*_ can be enhanced/redirected by coupling with modes of the plasmonic nanostructure at **r**.^24,25^ β(**r**) is the volume density of probability of the nanoemitter’s presence at **r** within *dV*, the elementary volume.

To optimize the manipulation of the light emission, β(**r**) must be controlled. In other words, the spatial distribution of the active medium must match the spatial distribution of the other elements of the HPN. Any misalignment such as that illustrated at the right side of Figure 1a is expected to jeopardize the weak coupling. In order to illustrate this point, let us consider an example extracted from ref (4). A gold bowtie was used for controlling both the excitation and emission of molecules (Figure 2b). In this article, a high enhancement factor of 1,340 on the fluorescence of a single molecule was reported. The molecules were randomly distributed and oriented near the bowtie, and in this configuration β(**r**) was not controlled, while the spatial distribution of the exciting near-field at 780 nm is confined within the gap (Figure 2c). As a result, only a few molecules did participate in the demonstrated effect. Additionally, only molecules oriented roughly parallel to the gap near-field (along the bowtie longitudinal axis) took advantage of the enhancement of the fluorescence via γ_exc_ (antenna effect for excitation). In particular, in Figure 2b, molecule 1 has a high chance to interact with the bowtie gap mode, while molecule 2 has not a suitable orientation (because the gap mode is mainly polarized parallel to the bow tie long axis), even though it is within the mode volume. As far as molecule 3 is concerned, it is not in interaction with the gap mode at all. A statistical study involving more than 200 molecules was done in this work. In other words, although the configuration shown in Figure 2b enabled the demonstration of important physical effects, the latter could be further enhanced if both the position and orientation of the molecules could be fully controlled. Besides, the background noise coming from noncoupled emitters (that are highly predominant) may constitute a serious limitation to achieve good contrast for light emission.

### Case of Strong Coupling (Figure 1b)

This regime of coupling differs from the previous one as in the case of strong coupling, the involved damping rates are lower than the energy transfer rate between the metal nanoparticle and the emitter/absorber.^26,27^ As a result, this energy can transfer back and forth between the two systems, creating a coupled hybridized system (named “polariton”) where it is no longer possible to tell the difference between the two separate systems. Such a strongly coupled system is characterized by Rabi oscillations (for a damped oscillating system),^7^ spectral splitting, and anticrossing in the dispersion function.^28^ This specific coupling regime generally affects the scattering spectrum,^29,30^ the absorption spectrum,^31^ and thus the extinction spectrum (absorption + scattering), but it can also tailor the emission spectrum^32,33^ through the spontaneous emission from the hybrid nanosystem.^34^ So, although photoluminescence has not always been systematically measured on strongly coupled nanosystems, hybrid plasmonic nanoemitters can also be adapted and optimized via the strong coupling regime.

Figure 1b illustrates the strong coupling regime between the two oscillators/resonators. Oscillator 1 is the plasmonic oscillator characterized by the eigenfrequency ω_1_^0^ including the effective mass *m*_1_ and the stiffness constant *k*_1_: ω_1_^0^ = (*k*_1_/*m*_1_)^1/2^. Oscillator 2 (eigenfrequency ω_2_^0^, damping γ_2_, mass *m*_2_, stiffness constant *k*_2_, ω_2_^0^ = (*k*_2_/*m*_2_)^1/2^) is the oscillator representing the active medium: molecular oscillator, excitonic oscillator. Both oscillators are coupled to each other via *g*, the coupling constant that has the unit of a frequency. The theory of strong coupling described, for example, in chapter 7.8 of ref (35) can be based on the classical motion equation of oscillators. Two coupled equations of motion are classically used for describing the strong coupling regime^36^ (eq 4):
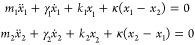
4where *x*_1_ and *x*_2_ are the positions of the oscillating entities (electrons, excitons, ...) and κ is the effective stiffness constant of the coupling. These equations do not contain any driving force. The problem thus consists of the determination of eigenmodes of the coupled oscillations having the form *x* = *x*_*p*_^0^*e*^*iω*∓*t*^, where *p* = 1,2 and ω_∓_ are the new eigenfrequencies of the coupled system.

We note that ω_*p*_^2^ = (κ + *k*_*p*_)/*m*_*p*_; κ/*m*_*p*_ = ω_*p*_*g*. In the case where *m*_1_ ∼ *m*_2_; *k*_1_ ∼ *k*_2_; ω_1_ ∼ ω_2_ ∼ ω_0_ ∼ ω; and ω^2^ – ω_0_^2^ ∼ 2ω_0_Δ, where Δ = ω – ω_0_, eq 4 becomes
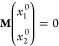
5, where **M** is a 2 × 2 matrix given by
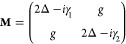
6

The cancellation of the determinant of **M**, corresponding to nontrivial solution for Δ, leads to a second-order equation (eq 7) allowing the determination of ω_∓_.
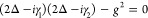
7The nature of the coupling between the two oscillators depends on the sign of the discriminant of eq 7 on which the nature of the solutions ω_∓_ depends. The condition 2*g* > |γ_1_ – γ_2_| results in damped (Rabi) oscillation with Rabi frequency of ≈2*g* corresponding to the spectral splitting (strong coupling), while condition 2*g* < |γ_1_ – γ_2_| leads to a weak coupling, resulting in a single decay with no oscillation.

In other words, in principle, a given coupled system can enter either the weak or strong coupling regime depending on the value of *g* relative to the damping rates. This interesting duality was highlighted in a recent review on the topic^26^ and in ref (7). As an interesting analogy, it is worth mentioning that the existence of two regimes, which can alternate with each other depending on the damping, is also observed in a classical RLC electric circuit based on energy transfer between inductance and capacitor through a resistor that induces a damping.^37^

For achieving this coupling, the two oscillators illustrated in Figure 1b must be side by side and align along the same direction.

From eq 4, it turns out that the necessary correlation between the coordinates x_1_ and x_2_ cannot be achieved if the relative position between the two oscillators is not controlled.

This is why the control of the spatial distribution and the orientation of oscillator 2 relative to oscillator 1 is of paramount importance in permitting and optimizing this coupling. This spatial control has a high influence on the value of *g*. The misalignment of the resonators, illustrated on the right side of Figure 1b, is expected to even cancel out *g* with no coupling. As a result, in ref (38), which deals with strong coupling within an ultrathin plasmonic gap, it was pointed out that “The nanoscale spatial position of the emitter and orientation of its dipole moment is crucially important since the local fields of nanogap structures can vary significantly across ∼10 nm and couple preferentially to one orientation of the dipole”. This quote illustrates well the problematic.

## Different Approaches Developed to Address the Issue of How to Control the Positioning of the Nanoemitter/Absorber

By searching for articles on the topic concerning “plasmons and weak coupling” or “plasmons and strong coupling”, we found that, since 2011, more than 4000 articles have been published on the subject with an increasing trend, whereas only about 600 articles are referenced for the period 2000–2010, which illustrates the interest of the topic. Several experimental approaches have been developed to control and optimize the spatial distribution of nanoemitters/absorbers in the vicinity of plasmonic antennas. The following section reviews some of the most significant approaches. Nine different approaches were identified and are being developed. Our review completes the numerous reviews about weak and strong coupling regimes^10−15,26,27,36^ (where the positioning issue was evoked but skimmed over) and another recent review dealing with deterministic integration of single solid-state quantum emitters with photonic nanostructures.^39^ Throughout this paper, the term “QD” (Quantum Dot) will be used to denote an inorganic colloidal semiconductor nanocrystal.

### Approach 1: Random Integration

Figure 2b illustrates this approach. A large number of reported works so far have relied on randomly positioned nanoemitters/absorbers such as molecules or nanocrystals that were typically first dissolved or suspended in a solvent or a PMMA (poly(methyl methacrylate)) solution before being spin-coated on or underneath plasmonic structures made by lithographic methods or by chemical synthesis. As another example, single silver (Ag) nanoprisms were deposited on a thin layer of cyanine dye molecules that form J-aggregates, resulting in a strong coupling at the single nanoparticle level.^40^ Efficient plasmon-molecular exciton strong coupling was demonstrated with an emitter containing metal–organic framework film that was homogeneously grown by layer-by-layer by spray coating on plasmonic nanoparticle lattices.^41^ Efficient strong coupling between 2D CdSe nanoplatelets (NPLs) and silver nanocubes was also achieved.^42^ A giant Rabi splitting energy up to 400 meV under ambient conditions was observed not only in scattering but also in the photoluminescence spectra. Although deposition of NPLs was randomly made on substrate, good conditions of NPL localization were found to demonstrate the effects.

As a last example, the use of wet-chemically synthesized single gold nanorods enabled fluorescence enhancement of weak emitters with a factor of 1100.^43^ In this research, gold nanorods were immobilized on a glass surface and covered with a solution of crystal violet (triphenylmethane) molecules in glycerol. The molecules randomly diffused in the nanorod’s near-field, without any control of their positions. Hence, only a small ratio of emitters falls randomly within the area of interest, coupled with the metal nanostructure.

### Approach 2: Nanospacer Engineering

**Figure 3:**
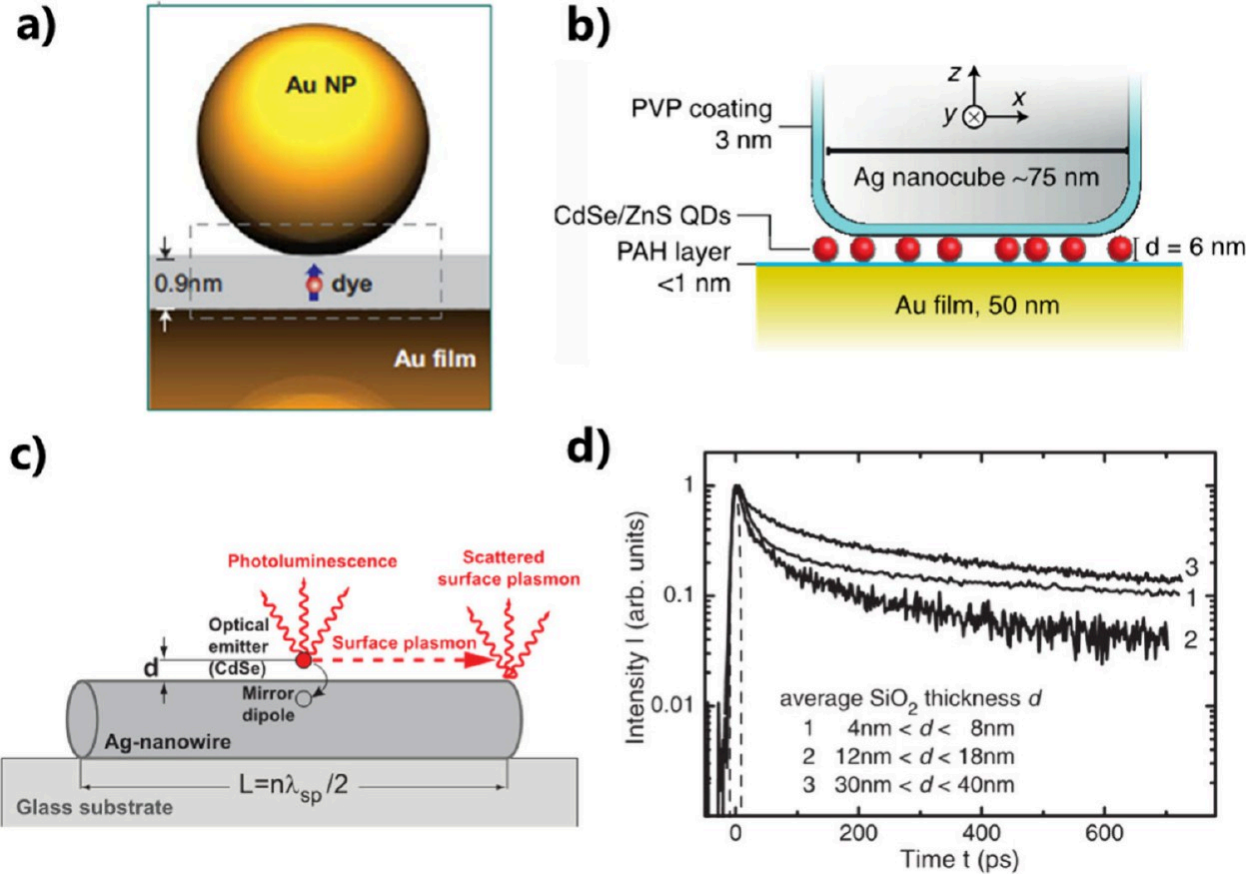
Control of nanospacers. (a) A single molecule trapped within a gap between a gold film and a gold nanoparticle. Adapted with permission from ref (30). Copyright 2016 Springer Nature Limited. (b) CdSe/ZnSe QDs trapped within a gap between a gold film and a silver nanocube. Reproduced with permission from ref (51). Copyright 2015 The Author(s) licensed under a CC-BY Creative Commons Attribution 4.0 License. (c) CdSe QDs coupled to a SiO_2_ coated silver nanowire, with controlled QD-nanowire distance and (d) PL lifetime for different gap thicknesses. Adapted with permission from ref (55). Copyright 2007 American Physical Society.

Some studies aimed at controlling the interaction between emitters and plasmonic cavities with a focus on ultrathin gaps that contain nanoemitters. In particular, nanoparticle-on-mirror structures were widely considered to achieve nanoscale plasmonic gap modes.^44,45^ The control of the gap thickness can be achieved by inserting a spacer layer. This issue was initially tackled by self-assembled monolayers, graphene, or molecular linkers to control the distance between a gold film and metal nanoparticles, enabling the study and optimization of localized light in subwavelength regions.^46−48^ By utilization of a 0.9 nm molecular spacing, individual molecules of methylene blue were strategically placed within the gap between gold nanoparticles and a gold mirror underneath. This configuration led to a strong coupling between the plasmonic cavities and a few molecules, even single molecule (Figure 3a).^30^

Furthermore, by using a homogeneous QD-containing solution at low concentration, few and single QDs were successfully integrated and resonantly coupled to a well-controlled plasmonic gap mode (Figure 3b).^49−51^

With nanoemitters brought within the gap, large fluorescence enhancement and strong coupling at the single emitter level were achieved in many studies.^52,53^ Nanospacers also enabled the control of metal-emitter distances, with no plasmonic cavity involved.^54,55^ An example is illustrated in Figure 3c with a Ag-nanowire (AgNW) cavity coupled with CdSe nanocrystals.^55^ A good control of the thickness *d* of the SiO_2_ layer separating the nanowire from the CdSe QDs led to a control of both radiative and nonradiative de-excitation of the QDs. In particular, their emission dynamics, related to the PL lifetime, could be controlled (Figure 3d). Some other reported works used quantum dots as both emitters and spacers. For example, in ref (56), colloidal silicon (Si) quantum dots, 3 nm in diameter, were successfully sandwiched between gold nanorods and a gold mirror, imposing a 3 nm thick gap. Although the Si QDs were homogeneously deposited on the gold mirror by drop casting before the deposition of nanorods, the observed enhancement factor, with respect to the luminescence from a monolayer of QDs on a flat gold film, reached a factor of 900. This effect illustrates a specificity of the use of nanocavities based on ultrathin dielectric gaps/nanospacers: the nanoemitters trapped in there benefit from the strong hybridized gap mode while the nanoemitters outside the gap lead to a very weak photoluminescence background. This effect thus enables a sort of “spatial self-selection” of the nanoemitters.

These systems, based on a designed nanospacer, are relatively simple to fabricate and offer precise control over the emitter properties. However, despite the interesting specificity mentioned above in the case of the plasmon nanocavity, this approach is limited to placing the emitter within the gap region perpendicular to the metal surface. It does not provide exact control over the emitter’s in-plane position within the gap.

### Approach 3: Lithography Carried out around Preidentified Nanoemitters

**Figure 4:**
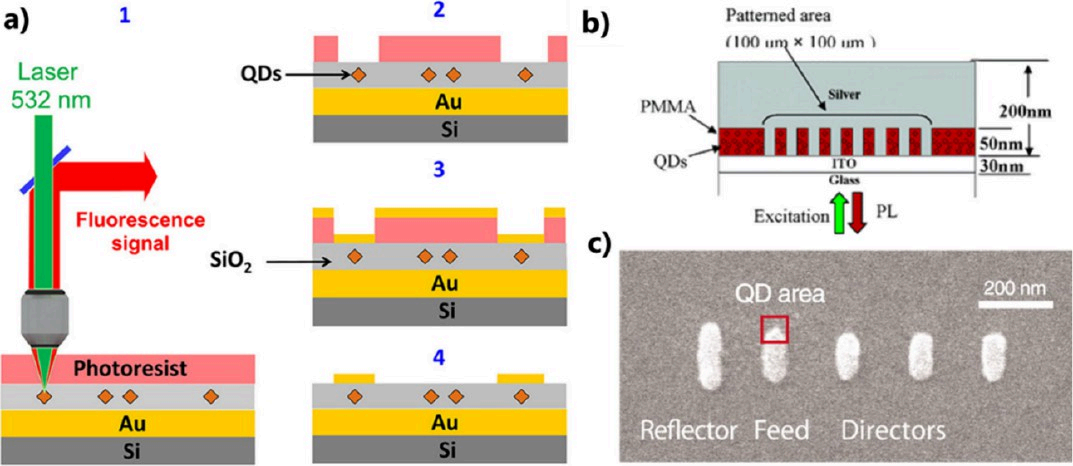
Use of optical or electron-beam lithography. (a) Deterministically positioning of an identified single QD within a patch antenna that is made around the QD by optical lithography. Reprinted with permission from ref (63). Copyright 2013 American Chemical Society. (b) Patterning by EBL of a silver array within QD-containing PMMA solution. Reprinted with permission from ref (64). Copyright 2005, American Chemical Society. (c) EBL-defined local functionalization on a Yagi-Uda plasmonic antenna, enabling deterministic integration of a single QD, adapted with permission from ref (24). Copyright 2010 The American Association for the Advancement of Science.

In situ optical and electron beam lithography (EBL) techniques have enabled the precise positioning of plasmonic antennas in the immediate vicinity of small groups or a few colloidal QDs with a nanometric accuracy.^57^ This approach consists in optically identifying the spatial localization of deposited QDs through photoluminescence mapping. Subsequently, a lithographic process is used to fabricate the plasmonic cavity in the vicinity of the identified QD, in situ, while measuring the quantum dot photoluminescence. This approach was also used for coupling single nanoemitters to monolithic microlenses,^58^ circular dielectric gratings,^59^ polymer waveguides,^60^ and Bragg pillar microcavities.^61,62^ It does mean that the accuracy of defining the photonic structure around the QDs depends on the resolution of the photoluminescence setup. Figure 4a shows an example where clusters of CdSe QDs were sandwiched by a gold patch antenna fabricated by optical lithography using a positive-tone photoresist followed by a lift off process.^63^ Shifting the cluster within the laser spot at the nanometer scale to maximize its fluorescence intensity achieved cluster centering with an accuracy of ±25 nm. Thus, using far-field optical lithography, a single quantum dot was successfully positioned on micrometer size patch-antennas with a 25 nm accuracy. The same approach was employed to create periodic silver arrays from a QD-containing PMMA layer using EBL (Figure 4b).^64^ Notably, this technique does not require the final lift-off step, which typically removes the PMMA. The major challenge in this process is to preserve the properties of the colloidal QDs. In a complementary approach to the one used in ref (63), an in situ far-field laser etching lithography technique was utilized. This technique allowed for the precise placement of single CdSe/CdS core–shell QDs within a subwavelength plasmonic patch antenna, achieving a remarkable 3 nm vertical and a 50 nm lateral precision.^65^

### Approach 4: Selective Surface Functionalization by EBL

EBL was also employed to deterministically attach QDs to plasmonic antennas in a two-step approach. In the first step, antenna structures were fabricated on an indium tin oxide (ITO) substrate. In the second step, a specific area for chemical binding of the QDs was defined by covering the metal antenna with PMMA. Local electron irradiation, followed by resist development, created nanoholes in the resist, which allowed for the exclusive formation of a self-assembled monolayer of mercaptoundecanoic acid. Subsequently, functionalized core–shell QDs can get immobilized on the functionalized areas, and the removal of the resist reveals QDs selectively attached to specific sites of the antenna. This powerful method enabled the precise integration of QDs on a Yagi-Uda plasmonic antenna^24^ (Figure 4c) or on a dimer antenna.^66^ The resulting advanced HPN presented highly directional emission and fluorescence enhancement.

It is worth pointing out that, in general, all the techniques based on electronic or optical beam lithography are very interesting to fabricate a few samples for a proof of concept and fundamental studies, but they cannot really be foreseen for any practical applications.

### Approach 5: Using Driving Forces

**Figure 5:**
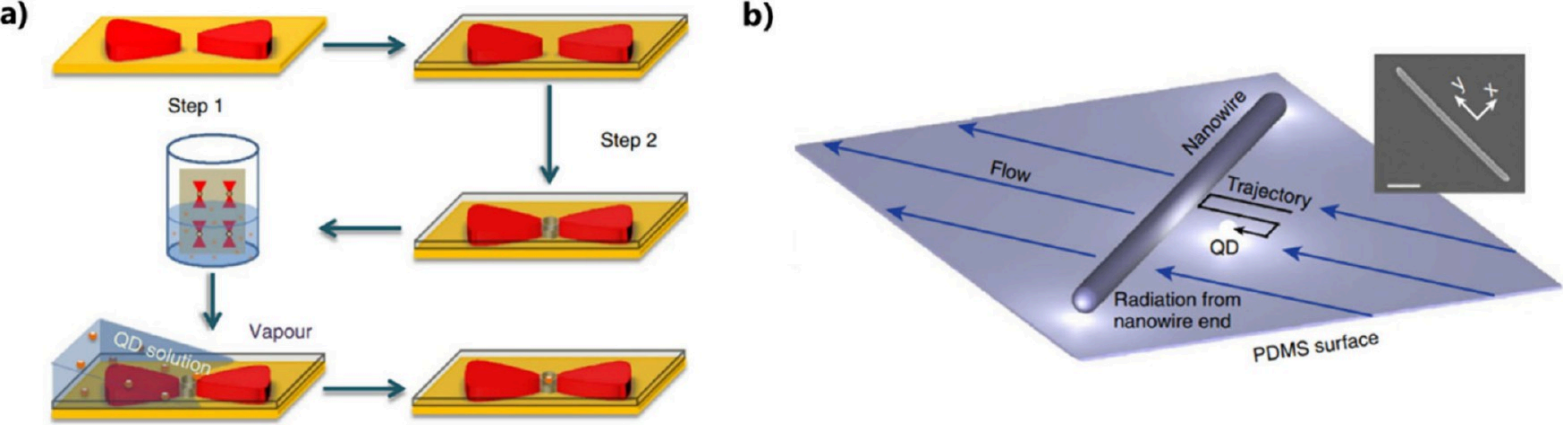
Positioning techniques based on driving capillarity forces. (a) Schematic illustration of the two-step lithography process for making holes at the center of bowtie structures and the interfacial capillary force assisted method for driving QDs into the holes. Adapted with permission under a Creative Commons CCBY 4.0 License License from ref (68). Copyright 2016 The Author(s). (b) A single QD is driven along a trajectory close to the wire by flow control. The inset shows a scanning electron microscopy image of a typical Ag nanowire used in these experiments (scale bar: 1 μm). The x–y coordinate system is defined relative to the orientation of the nanowire, as illustrated in the inset. Adapted with permission from ref (69). Copyright 2013 The Author(s).

An alternative method for positioning QDs at desired locations involves creating hollow apertures and using capillarity forces to direct the nanocrystals into patterned holes.^67,68^ As illustrated in Figure 5a, QDs were trapped in lithographically patterned holes within bowtie gaps using interfacial capillary forces.^68^ This approach enabled the strong coupling between the bowtie cavity and the QDs at the single quantum emitter limit. Another example, illustrated in Figure 5b, involved confining QDs to a thin sheath along the surface through the fluid chemistry in a microfluidic device through electro-osmosis.^69^ This process allowed the movement and positioning of QDs at desired locations onto an AgNW via viscous drag. In this experiment, QD positions were tracked with an accuracy of 12 ± 1 (11 ± 1) nm along the *x*(*y*) directions and positioned with a precision of 34 ± 3 (39 ± 3) nm.

Optical forces controlled at the nanoscale were also used to position nanoemitters. Plasmonic tweezers based on spatially confined attractive gradient forces were used to manipulate fluorescent nanoparticles to trap them in the vicinity of Ag nanodisks on SiO_2_ pillars.^70^ This approach has the advantage of using specific polarization states of light for manipulating the nanoemitters.

### Approach 6: Using Scanning Probe Microscopy

**Figure 6:**
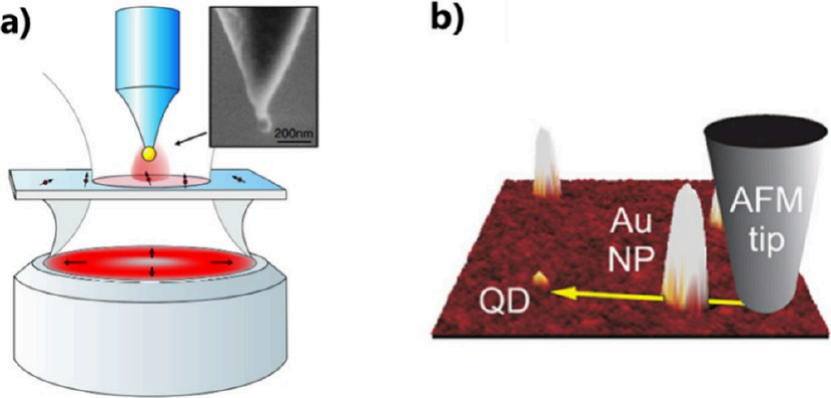
Using scanning probe microscopy. (a) Sketch of an experimental arrangement of a SNOM. Inset: SEM image of a gold nanoparticle attached to the end of a pointed optical fiber. Adapted with permission from ref (71). Copyright 2006 American Physical Society. (b) AFM image of a QD and Au NPs with a yellow arrow denoting the path of the gold nanoparticle controlled by an AFM probe. Adapted with permission from ref (73). Copyright 2011 American Chemical Society.

Another approach relies on the use of scanning probe microscopy for coupling nanoemitters to plasmonic antennas. An early work developed an advanced tip for scanning near-field optical microscopy (SNOM).^71^ A spherical Au nanoparticle was integrated at the end of a pointed etched optical fiber to act as a nanoantenna which could be positioned in all three directions with a nanometer precision using piezo stages. This method enabled the investigation of different regimes of interaction between the Au nanoparticle and single molecules of nile blue deposited on a substrate (see Figure 6a as an illustration). In particular, both fluorescence enhancement and quenching were observed. In a similar example, an aluminum bowtie antenna was fabricated at the apex of Si_3_N_4_ atomic force microscopy (AFM) tip by focused ion beam milling.^72^ The interaction of a single quantum dot with the bowtie antenna was controlled as when scanning the probe above the quantum dot, its photoluminescence was enhanced while its excited-state lifetime decreased. Besides, the AFM was used for nanomanipulation. A single Au nanoparticle was controllably pushed close to a nearby CdSe/ZnS QD.^73^ The coupling between the two particles turned out to vary in a systematic and reversible manner (Figure 6b).

### Approach 7: Selective Growth or Attachments at Pointed Apexes

**Figure 7:**
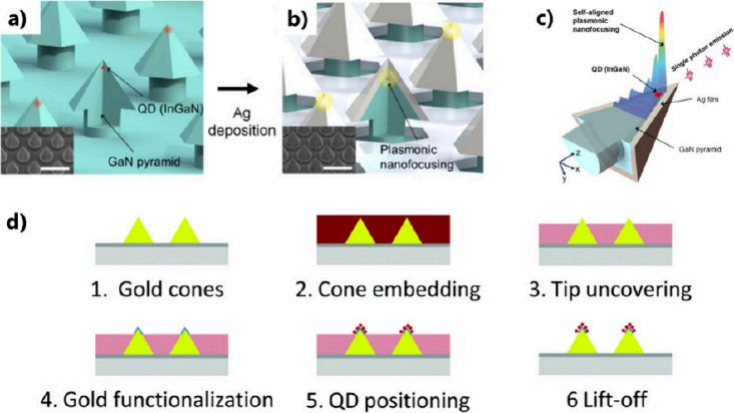
Selective growth and functionalization at pointed apexes. (a) Schematic of the site-controlled InGaN QD in the GaN nanopyramid structure. Red islands indicate single InGaN QDs, while the green region is an InGaN quantum well. (b) Structure after silver deposition. The scale bars in the SEM images (insets) represent 500 nm. (c) Plasmonic nanofocusing self-aligned with the QD. (a–c) Adapted with permission from ref (74). Copyright 2015 National Academy of Sciences. (d) Different steps for selective attachment of single QDs on top of gold nanocones. Reproduced with permission from ref (77). Copyright 2015 The Royal Society of Chemistry.

Several articles have reported on methods for precisely positioned QDs by selective growth in various tapered metallic structures such as pyramidal tips.^74−76^ As an example, Figure 7a–c provides the schematics of integrating single InGaN QD at the apex of silver-coated GaN nanopyramid structures.^74^ Hexagonal pyramidal GaN structures were first grown on a hole-patterned mask by metal–organic chemical vapor deposition (MOCVD). Subsequently, an InGaN single quantum well layer and a GaN barrier layer were grown on the GaN pyramid structures to achieve a single QD on top of the pyramid. Single QD naturally formed at the apex of each pyramid due to the three-dimensional confinement of the exciton (electron–hole pair) wave function. Finally, a 40 nm thick silver layer was directly deposited using an electon-beam evaporator.

As a result, the plasmonic modes are highly focused near each single QD formed on the pyramid apex, in a self-aligned fashion with lateral spatial accuracy better than 5 nm. Such structures enabled self-aligned plasmonic nanofocusing in the QD of the metal-pyramid hybrid structure, resulting in an InGaN QD emission with a 5000-fold two-photon luminescence intensity.^76^ This approach is an unprecedented method for single emitter–light coupled devices and an essential technology for on-chip quantum devices. However, this technique is limited to tapered pyramidal nanostructures and cannot be extended to, for example, chemically synthesized emitters and nanostructures with strong potential for inexpensive and flexible applications. Another elegant approach for selective QD integration based on the exploitation of the apex of pointed nanostructures was proposed in ref (77) (see Figure 7d). Gold nanocones were first fabricated on a glass substrate by EBL. The cones were embedded in a resist which was then spread by spin coating. As a result, the tips of the cones were not covered by the resist (scheme 3 of Figure 7d). This spatial selection is the result of fine-tuning the spin coating process and takes advantage of the geometry of the pointed gold cones. The uncovered gold surface of the cones was then functionalized using thiol chemistry, enabling the subsequent selective positioning of functionalized QDs on the cone tips. The removal of the resist leads to hybrid plasmonic nanoemitters presenting deterministically positioned QDs. This hybrid nanosystem, coupled with a circular Bragg antenna, enabled the demonstration of an ultrabright nanosource of high directionality, leading to an increase in the observed brightness by a factor as large as 800.^78^

As a final example of the use of a small radius of curvature, it was shown that sulfhydryl groups (also called thiol group −SH) preferentially functionalize the gold atoms at the sharper corners near the extremities of colloidal Au nanorods, where fewer surfactant molecules (cetyltrimethylammonium bromide CTAB) are adsorbed.^79^ This curvature effect made it possible to facilitate the binding of surface-functionalized QDs to these gold atoms, right at the nanorod’s ends where the plasmonic-enhanced electric field is at its maximum. This approach enabled the demonstration of a large spectral Rabi splitting for a single QD that is strongly coupled with the plasmons of the nanorod.

### Approach 8: DNA Origami

**Figure 8:**
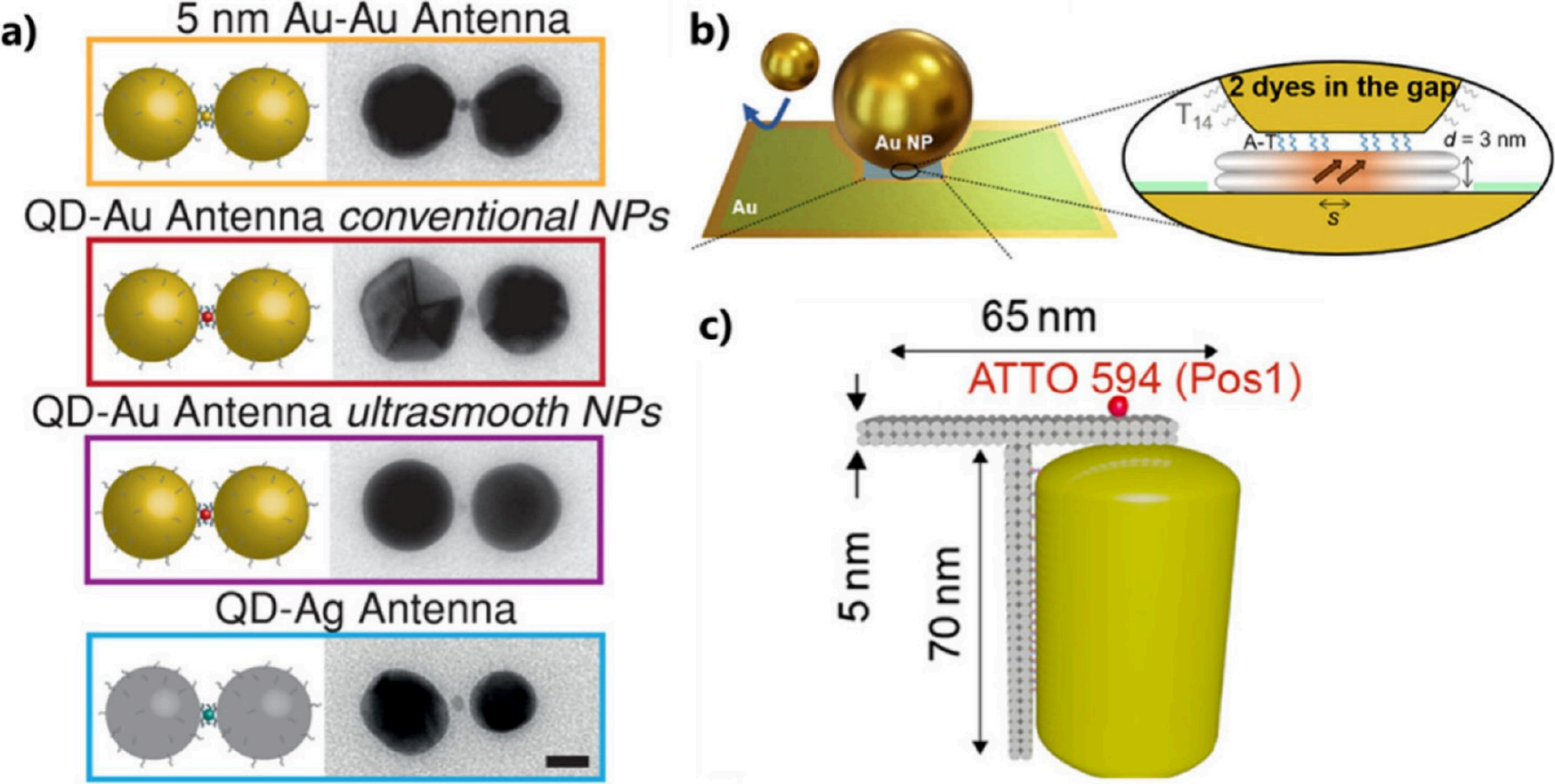
Use of DNA origami. (a) Scheme of gold or silver dimer antennas whose gaps host a gold nanoparticle (orange), or singles QDs (red or blue). Scale bar is 20 nm. Adapted with permission from ref (85). Copyright 2019 Wiley-VCH Verlag GmbH & Co. KGaA, Weinheim. (b) Assembly of nanoparticle-on-mirror cavities using DNA nanotechnology. Adapted with permission under a Creative Commons CC-BY4.0 License from ref (86). Copyright 2023 The Authors. (c) Sketch of a T-shaped DNA origami structure hosting a gold nanorod and a single dye molecule (red sphere) placed at the position Pos1 (Adapted with permission from ref (87). Copyright 2023 American Chemical Society).

DNA-based approaches are a powerful tool used to arrange optically active hybrid plasmonic nanocomponents with a high accuracy.^80−83^ We invite the reader to read a recent review on the topic.^80^ Using the addressability of DNA, it is viable to control the precise arrangement of several quantum emitters in optical nanocavities located between two gold surfaces. Using this approach, single molecules can be trapped within the gap of a dimer plasmonic antenna, enabling a 5000-fold fluorescence enhancement.^82^ Using a short transverse double-strand DNA, five dye molecules were trapped in the gap between two 40 nm gold particles, resulting in the achievement of the strong coupling.^84^ A straightforward approach to assemble plasmonic antennas, consisting of two 40 diameter metallic nanoparticles with a single colloidal QD positioned in the gap, at the hot spot, is illustrated in Figure 8a.^85^ Furthermore, DNA origami enabled the precise positioning of single-quantum emitters in ultranarrow plasmonic gaps, facilitating a detailed study of their modified light emission (Figure 8b).^86^ In addition to the positioning in small gaps, the nanometric control of the distance between an individual dye and a single colloidal gold nanorod was investigated using a T-shaped DNA origami structure. This origami design enabled the incorporation of a single fluorescent molecule at the tip of a gold nanorod, 5 nm away from the gold surface (Figure 8c).^87^ This DNA-based approach is elegant but does not provide high flexibility in nanoemitters positioning because it requires customized, additional chemical modification of the QD/nanostructure surfaces. Additionally, although the DNA-based hybrid nanoemitters can be dried, they are relatively fragile due to the need of a salty liquid environment for the survival of DNA origami, limiting the suitability of this approach for direct integration into functional nanophotonic devices.

### Approach 9: Plasmonic Photopolymerization

Plasmon-induced polymerization has also been used for integrating nanoemitters with a high spatial precision.^3,88^ Compared to the other above-described methods, this approach leverages the intrinsic electromagnetic modes of the metal nanostructures to drive the local integration of nanoemitters. Through one-or-two-photon processes, the plasmonic optical near-field is absorbed locally by photosensitive dyes, whose excited states form radicals that interact with a monomer (typically acrylate-type molecules) to trigger polymerization.^89^ As a result, polymer nanoparts are integrated on the nanoparticles, creating a 3D replica of the plasmonic modes.^90−95^ The hybrid structure is obtained after development, involving rinsing to remove any nonpolymerized liquid. The spatial distribution of the resulting polymer molds is related to the spatial distribution of the nanoparticle electromagnetic near-field radiation. The approach relies on the precise control of the energy threshold for the polymerization, which is the minimum energy required to trigger local polymerization. The incident energy is deliberately set below this threshold, ensuring nothing happens in the surrounding monomer formulation except within the nanoparticle near-field where light is locally plasmon-enhanced. The photopolymer can contain or support nanoemitters,^96−98^ enabling the development of advanced HPNs with various spatial anisotropies of the active medium.

It should be noted that this approach has been developed within the broader context of plasmon-induced nanochemistry,^99−101^ where localized surface plasmons can modify the environment of metal nanoparticles through local light, hot electrons, or heat. In addition to photopolymerization, photochemical reactions can encompass processes such as the reduction of diazonium salts,^102,103^ photolysis of PMMA,^104^ isomerization of azobenzene molecules,^105^ photochemical degradation of an oligomer shells,^106^ and reduction of metal ions.^107^ Plasmon-induced nanochemistry can potentially enable deterministic integration of nanoemitters on metal nanostructures.

The following sections delve into how the symmetry of the active medium in HPNs can be controlled through plasmon-induced photopolymerization of photosensitive materials containing or supporting quantum emitters.

## Focus on Approach 9: Control of the Spatial Symmetry of the Active Medium by Plasmonic Photopolymerization

In group theory, molecules or other nano-objects can be classified into point groups based on the type and number of symmetry operations they possess.^108^ This description was applied in nanoplasmonics to discuss, in particular, the properties of metal nanoparticles and artificial “plasmonic molecules” of different shapes.^109,110^ In the following, we use this nomenclature for describing the way plasmonic photopolymerization can enable the control of the spatial symmetry of HPNs. This section concerns exclusively the weak coupling regime.

### Exploiting Metal Nanoparticles of D_∞h_ Symmetry

**Figure 9:**
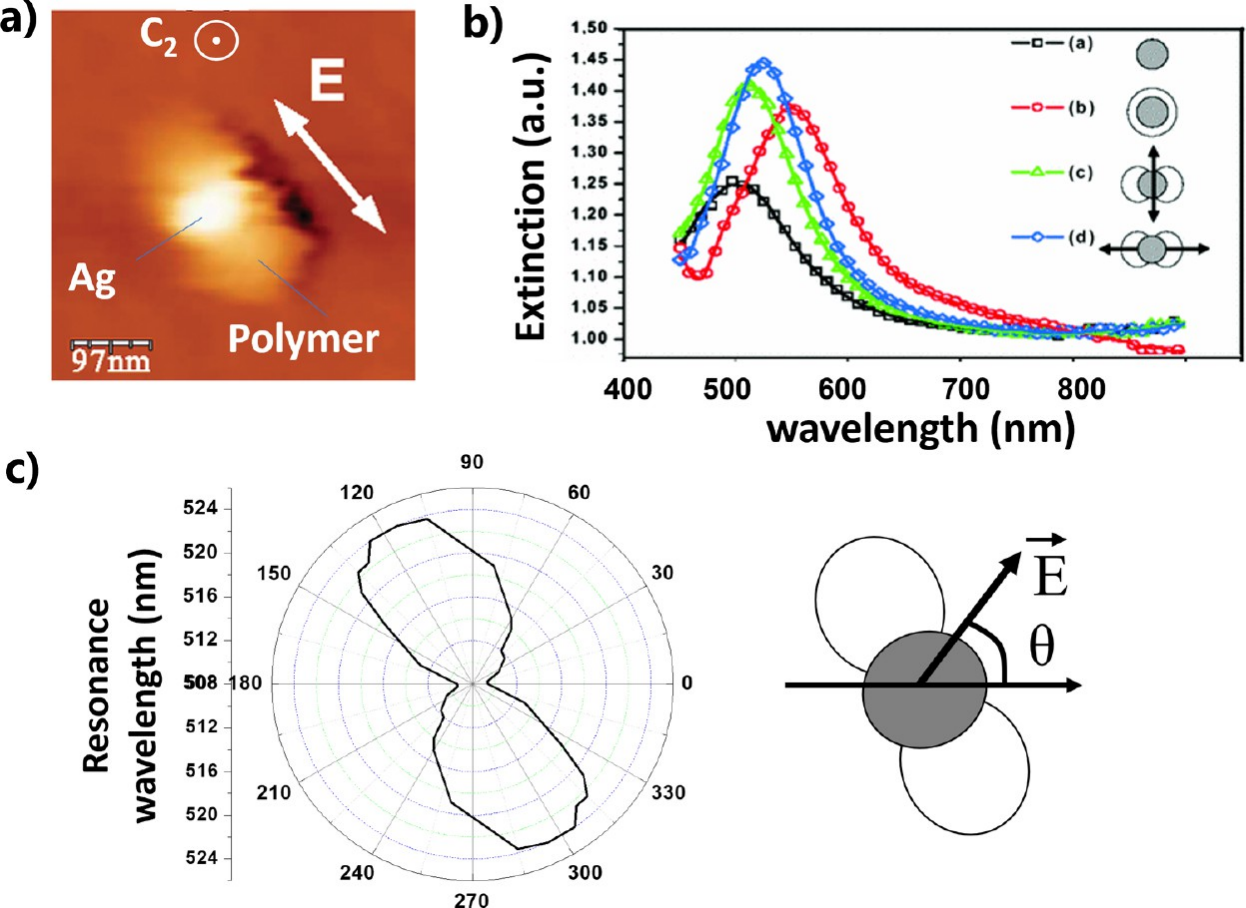
Plasmonic photopolymerization on a silver nanodisk. (a) AFM image of the hybrid nanosystem resulting from local polymerization induced by the dipolar plasmonic induced field at λ = 514 nm using an incident wave vector parallel to the *C*_2_ axis, and a linearly polarized field **E** represented by the white arrow. (b) Extinction spectra of arrays of different kinds of hybrid plasmonic structures (see text for details). (c) Polar diagram showing the wavelength of plasmonic resonance of an array of hybrid particles like that of figure (a), as a function of the angle of polarization θ of the white light used for extinction spectroscopy (represented on the right). (a–c) Adapted with permission from ref (90). Copyright 2007 American Physical Society.

The possibility to structure the dielectric environment of metal nanoparticles using their intrinsic plasmonic field opened new avenues for controlling the symmetry of the active medium of HPNs. One of the earliest demonstrations of symmetry modification at the nanoscale was reported in 2007 in ref (90) where the authors used the dipolar plasmonic field of silver nanodisks excited with a linearly polarized field (at λ = 514 nm) to trigger a one-photon polymerization reaction in the vicinity of the nanodisks. As a result, an anisotropic polymer nanostructure, like an ellipsoid of indexes, was made at the surface of the nanodisks (see Figure 9a). The process led to a modification of the local symmetry of the surrounded dielectric medium: the initial *D*_*∞h*_ symmetry of the nanodisks was transformed into a *D*_2*h*_ symmetry nanosystem where the structure can be retrieved after a π-rotation around the *C*_2_ axis perpendicular to the object plane.

This decrease of the degree of symmetry led to a spectral degeneracy breaking of the nanodisk’s plasmon resonance (Figure 9b,c). The bare Ag nanodisk on a glass substrate presents a single peak of resonance (Figure 9b, curve (a)). This peak remains unique and undergoes a red-shift when the nanodisk is immerged in the liquid formulation before the exposure (Figure 9b, curve (b)). This isotropic immersion keeps the *D*_*∞h*_ symmetry (not considering the substrate). The hybrid anisotropic nanosystem made by plasmonic photopolymerization becomes polarization sensitive. It presents two new eigenmodes: one perpendicular to the integrated polymer nanolobes (Figure 9b, curve (c)) and one along the lobes (Figure 9b, curve (d)). This induced polarization sensitivity is illustrated in Figure 9c (left), which displays a polar diagram of the plasmon resonance peak as a function of polarization angle of the incident white light, as represented on the right of Figure 9c.

It is worth noting that when the metal nanoparticle is illuminated with a wave vector perpendicular to the substrate on which it is deposited the symmetry problem becomes simplified: only in-plane symmetries around the out-of-plane C-axes need to be considered. In particular, *D*_*nh*_ symmetry groups (where n is an integer) can be described by subgroups *C*_*nv*_. This simplification can be applied to all of the cases discussed in the article.

**Figure 10:**
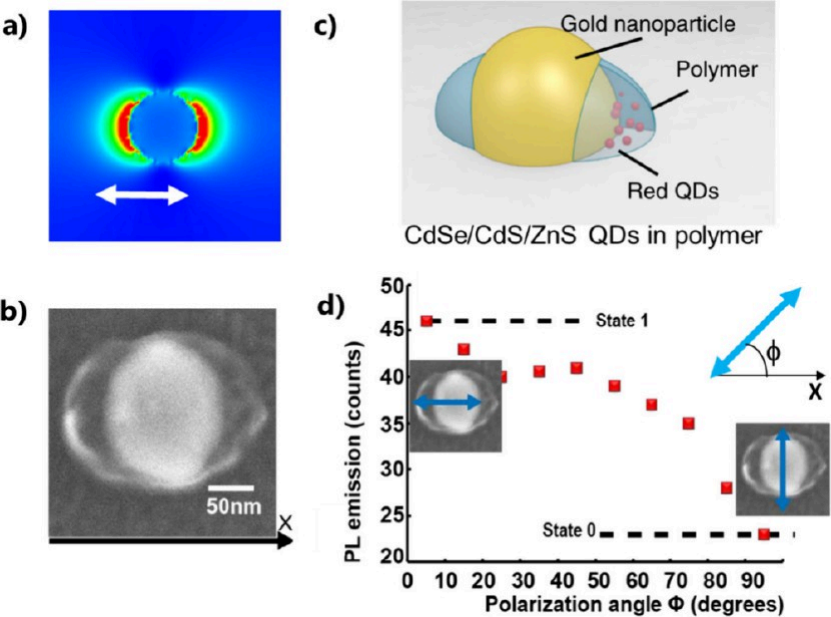
*D*_∞*h*_ symmetry of a gold nanodisk leads to *D*_2*h*_ symmetry of the active medium by Plasmonic two-photon polymerization (P2PP) using linear polarization. (a) FDTD calculation of the intensity of the near-field, used for P2PP, of the nanodisks at λ = 780 nm. The white arrow represents the direction of polarization used for P2PP. (b) SEM image of the resulting HPN: Au nanodisk surrounded by two polymer lobes containing QDs. (c) Illustration of the hybrid nanosystem. (d) Photoluminescence intensity from the hybrid structure as a function of ϕ; the angle of polarization of the exciting linearly polarized laser beam at 405 nm wavelength. The figure on the right represents the *X*-axis, ϕ, and the linear polarization used for post characterization. (a–d) Adapted with permission from ref (3) under Creative Commons CC BY license. Copyright 2020 The Author(s).

This control of the symmetry was further exploited for the development of advanced HPNs. The experiment from ref (90) was reproduced using gold nanodisks and a photopolymerizable formulation containing semiconductor colloidal CdSe/CdS/Zn QDs.^3^ The dipolar plasmon mode excited with *X* polarized light at λ = 730 nm (Figure 10a) was employed to initiate a local photopolymerization reaction, following two-photon absorption. This process is known as Plasmonic 2-Photon Polymerization (P2PP).

**Figure 11:**
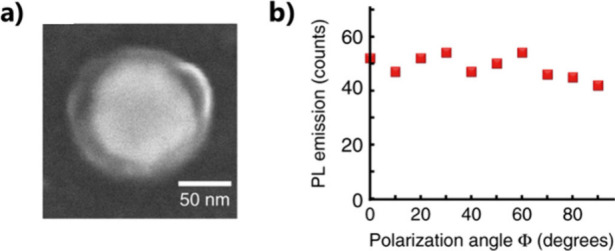
*D*_∞*h*_ symmetry of a gold nanodisk transferred to the active medium by P2PP using circular polarization. (a) SEM image of a gold nanodisk surrounded by a polymer ring containing QDs. (b) Photoluminescence from the hybrid structure, excited with a linearly polarized laser beam at 405 nm wavelength, as a function of the polarization in-plane angle ϕ. (a, b) Adapted with permission from ref (3). Copyright 2020 The Author(s) under Creative Commons CC BY license.

The resulting HPN is shown in Figures 10b,c. It exhibits *C*_2*v*_ symmetry of the active medium, primarily confined along the *X* direction. When excited by linearly polarized blue light at 405 nm (a wavelength well absorbed by QDs), it demonstrates an intensity of photoluminescence that strongly depends on the direction of the incident polarization (Figure 10d). In particular, higher PL is observed with an incident polarization parallel to the QD-containing polymer nanolobes along *X*. This polarization sensitivity was found to result from the nanoscale anisotropy of the active medium and was interpreted in terms of a spatial overlap between the active medium and the local exciting field, as quantified by the overlap integral:^3^
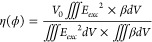
8where *E*_*exc*_(*x*,*y*,*z*) is the amplitude of the local field that excites the QDs at 405 nm of wavelength. Although the Au nanodisk is not resonant at this wavelength, *E*_*exc*_ is local, spatially anisotropic, and maximum along the incident polarization.^3^ β(*x*,*y*,*z*), introduced in eq 3, is the volume density of probability of presence of the nanoemitters. Since the latter are contained in the polymer nanoparts, β(*x*,*y*,*z*) can be determined by SEM or AFM imaging of the polymer. *V*_0_ is a constant that is homogeneous to a volume and can be considered as the total volume of integration. While β(*x*,*y*,*z*) has a fixed spatial distribution for a given HPN, the map of *E*_*exc*_(*x*,*y*,*z*) varies with ϕ, the angle of polarization, enabling the modulation of the spatial overlap between *E*_*exc*_ and β, thus allowing one to tune the excitation of the QDs and the resulting PL intensity. β(*x*,*y*,*z*) can be designed by choosing the polarization state used for P2PP. In the case of Figure 10a,b, the orientation of the two lobes can be modified by adjusting the linear polarization direction of the incident light used for photopolymerization. Using other states of polarization for P2PP can lead to different symmetries. Figure 11 shows an example of an HPN obtained from a gold disk excited with circular polarization during the P2PP process. The resulting HPN (Figure 11a) maintains the initial *D*_*∞h*_ (*C*_2*v*_ considering the substrate) symmetry, making it insensitive to the incident polarization: the recorded PL level remains constant regardless of the angle of the linear polarization used to excite it (Figure 11b). In other words, the *D*_*∞h*_ symmetry of the nanodisks was transferred to the active medium.

**Figure 12:**
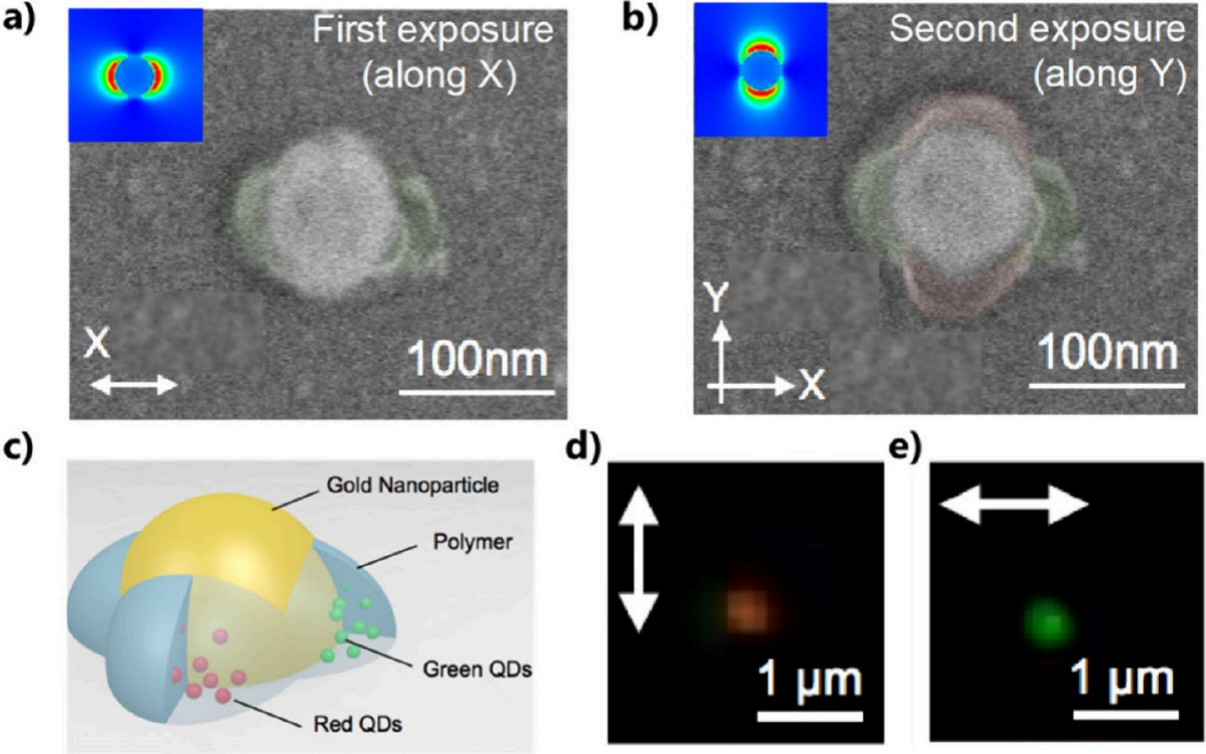
*D*_∞*h*_ symmetry of a gold nanodisk leads to *D*_4*h*_ symmetry of the active medium and a 2-color system by 2-step P2PP using linear polarization. (a) SEM image of HPN resulting from P2PP using *X*-polarized light: the gold nanodisk surrounded by two polymer lobes containing green QDs. (b) SEM image of the same HPN resulting from subsequent P2PP using *Y*-polarized light with another photopolymer formulation: the Au nanodisk surrounded by two pairs of polymer lobes along *X* and *Y*, containing green and red QDs, respectively. In (a), (b) the top left inserts are FDTD calculation of the intensity of the near-field, used for P2PP, of the nanodisks at λ = 780 nm. (c) illustration of the resulting hybrid nanosystem. (d, e) Far-field photoluminescence image from the hybrid structure shined at 405 nm wavelength by a laser beamlinearly polarized along *Y* and *X*, respectively. (a–e) Adapted with permission from ref (88). Copyright 2015 American Chemical Society.

The nanodisk’s *D*_*∞h*_ symmetry can be transformed into a *D*_4*h*_ (*C*_4*v*_ considering the substrate) symmetry through a two-step approach.^88^ The first step used a *X*-polarized light for P2PP. After development (Figure 12a), the P2PP was repeated with perpendicular *Y*-polarized light, resulting in a four-lobe HPN (Figure 12b). This two-step approach has a crucial asset: it allows for the use of different hybrid photopolymerizable formulations at each step. In particular, two formulations were employed: one containing green photoluminescent QDs and the other containing red QDs. In that way, a two-color system of *D*_4*h*_ symmetry, illustrated in Figure 12c, was created. This unique HPN presents two PL modes that can be selected with a linearly polarized excitation at 405 nm: PL is dominated by a green color for *X*-polarization (Figure 12e) and by a red color for *Y*-polarization (Figure 12d). This innovative polarization-driven two-color nanopixel relies on the control of the HPN’s symmetry through P2PP.

### Exploiting the D_4h_ Symmetry of Metal Nanoparticles

**Figure 13:**
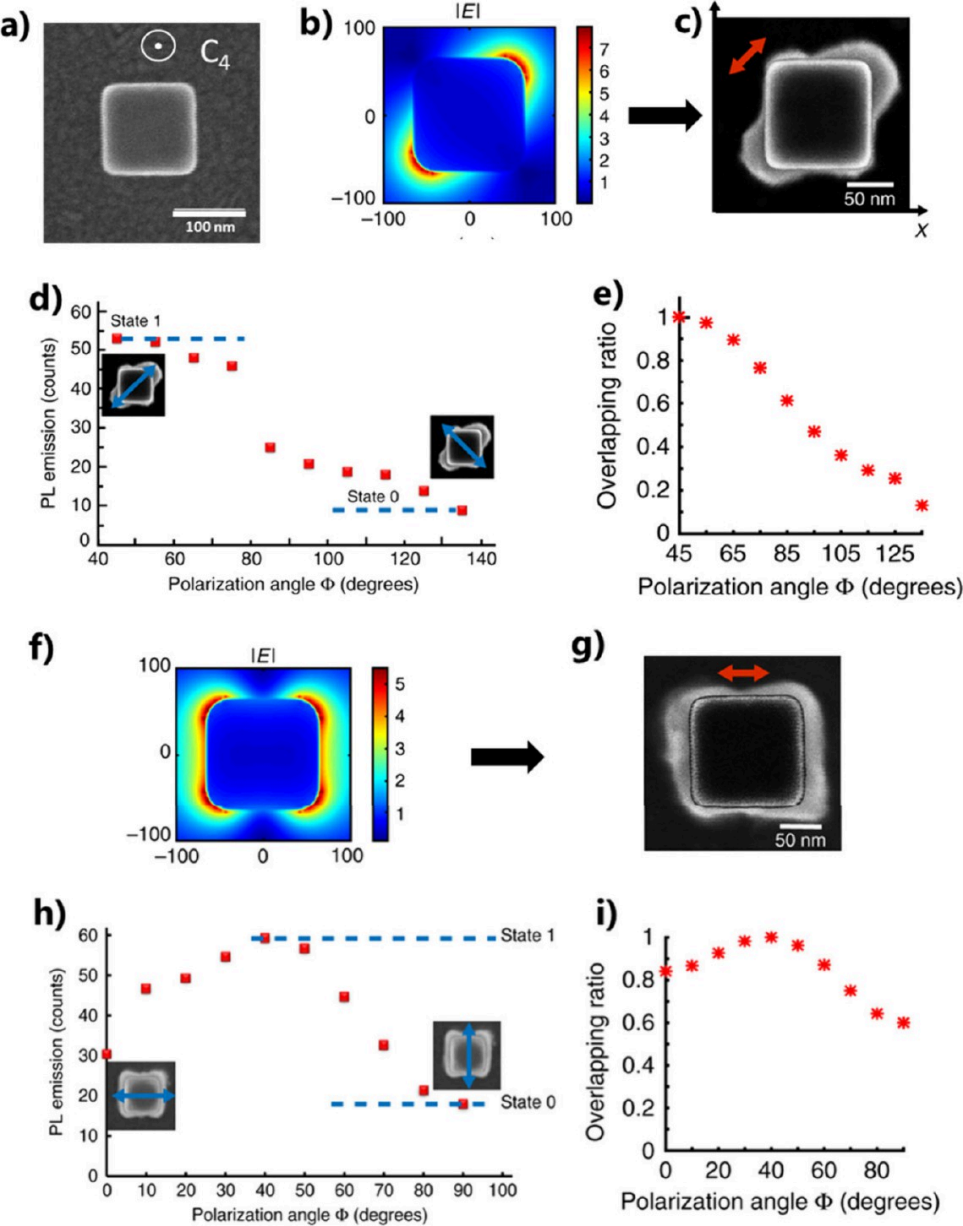
*D*_4*h*_ symmetry of a gold nanocube leads to *D*_2*h*_ or *D*_4*h*_ symmetry of the active medium by P2PP using linear polarization. (a) SEM image of a gold nanocube. (b) FDTD calculation of the plasmonic near-field amplitude of the nanocube at λ = 780 nm. The incident polarization is along one of the diagonals of the cube’s top square (dipolar plasmonic eigenmode). (c) SEM image of the resulting HPN: the Au nanocube is surrounded by two polymer lobes containing QDs, oriented along one of the diagonals of the cube’s top square. The red arrow represents the direction of polarization used for P2PP. The plasmonic field is molded by the P2PP photopolymer. (d) Photoluminescence intensity from the hybrid structure in (c) as a function of ϕ, the angle of polarization of the exciting linearly polarized laser beam at 405 nm wavelength. (e) Corresponding calculated overlap ratio defined in eq 8. (f) FDTD calculation of the plasmonic near-field amplitude of a 100 nm edge nanocube at λ = 780 nm for an incident polarization parallel to one of the nanocube’s edge. (g) SEM image of the resulting HPN: the Au nanocube is surrounded by polymer, containing QDs, presenting a *D*_4*h*_ symmetry. The red arrow represents the direction of polarization used for P2PP. (h) Photoluminescence intensity from the hybrid structure in (g) as a function of ϕ, the angle of polarization of the exciting linearly polarized laser beam at 405 nm wavelength. (i) The corresponding calculated overlap factor defined in eq 8. (a–c,f–i) Adapted with permission from ref (3). Copyright 2020 The Author(s) under Creative Commons CC BY license.

Nanoparticles belonging to the *D*_4*h*_ point group are interesting in terms of symmetry. In particular, a nanocube presents many axes and planes of symmetry, each associated with different modal properties and applications.^111,112^ An example of a gold nanocube is shown in Figure 13a. If the incident illumination is parallel to the out-of-plane *C*_4_ axis, the symmetry problem becomes simplified, and only the in-plane symmetry needs to be considered. Figure 13b shows a calculated near-field map of a nanocube illuminated by a plane wave propagating along the *C*_4_ axis (normal incidence) and linearly polarized along the top square diagonal (at λ = 730 nm). The two-lobe electromagnetic field represents a dipolar eigenmode of the Au nanocube. Using this field for P2PP led to the hybrid structure shown in Figure 13c. The resulting structure exhibits *D*_2*h*_ symmetry of the active medium, making it highly sensitive to polarization. Figure 13d shows indeed a strong polarization dependence of the PL with a contrast of 70%. The numerical calculation of the overlapping ratio η (ϕ) (Figure 13e) reveals a clear correlation between the photoluminescence and the nanoscale spatial overlap defined in eq 8. It should be pointed out that the PL contrast, albeit important, never approaches 100%. This issue was discussed in ref (3). The contrast seems to be limited by the PL background resulting from the incident exciting field, regardless of the incident polarization. In particular, the calculated intensity maps show that the exciting near-field radiation is never fully nil within the emitter-containing polymer, letting us expect a nonzero resulting PL. This PL background does not come from the metal photoluminescence whose quantum yield is negligible compared to that of the semiconductor nanocrystals. We showed this in the Supporting Information of ref (3) through experimental comparison between hybrid nanoemitters containing QDs and those with no QDs added into the photosensitive formulation (*i.e.*, hybrid polymer/gold nanoparticles used as a reference which does not contain any quantum nanoemitters).

It is possible to transfer the initial *D*_4*h*_ symmetry to the active medium by exciting the nanocube with a polarization parallel to a cube’s side for P2PP. The associated field at 730 nm results from a combination of two eigenmodes (Figure 13f). The resulting HPN (Figure 13g) presents the *D*_4*h*_ symmetry of the particle’s near field. The PL intensity as a function of polarization angle (Figure 13h,i) exhibits a characteristic feature of the *D*_4*h*_ symmetry: it reaches a maximum at ϕ = 45°, corresponding to an additional thickness of the active medium at this angle.

### Polarization-Sensitive Single Photon Nanoswitch

**Figure 14:**
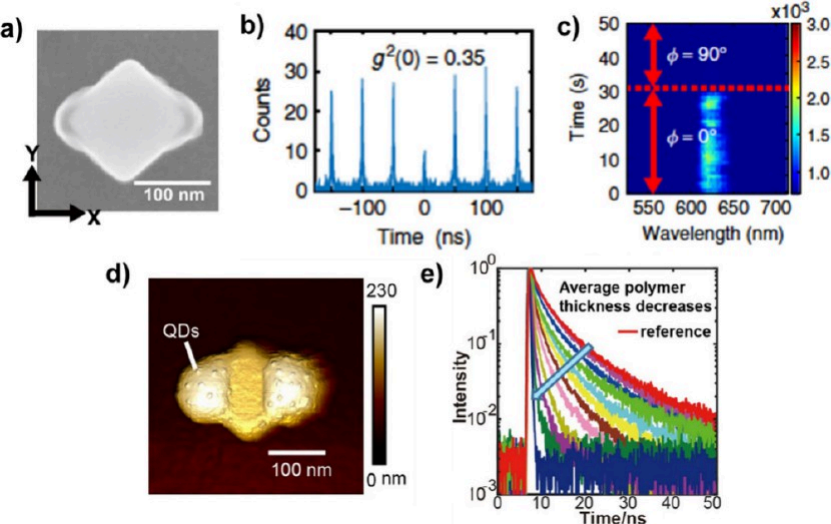
Advanced HPN based on a gold nanocube of *D*_4*h*_ symmetry. (a) Hybrid structure resulting from P2PP using X-polarized light at λ = 730 nm. The polymer lobes contain a single QD. (b) HBT *g*^(2)^ measurement revealing a single-photon emission (*g*^(2)^ (0) < 0.5). (c) Polarization sensitive single-photon nanoswitch. (d) AFM image of a hybrid HPN with functionalized polymer supporting QDs at its surface. (e) Lifetime measurements with different polymer thicknesses. (a–c) Adapted with permission from ref (3). Copyright 2020 The Author(s) under Creative Commons CC BY license. (d, e) Reproduced with permission from ref (98). Copyright 2022 Chinese Laser Press.

The polarization sensitivity of the *D*_2*h*_-symmetry hybrid structure in Figure 13c can be exploited at the single photon level. By reduction of the concentration of QDs within the initial photopolymerizable formulation, it became possible to trap a single QD in the vicinity of a corner of the gold nanocube. Figure 14a shows a nanocube-based hybrid structure containing a single QD and enabling single-photon emission, as shown by the well-known Hanbury Brown and Twiss (HBT) *g*^(2)^ correlation function measurement in Figure 14b, as a signature of a single photon source. Due to the HPN’s symmetry, the emission of single photon can be polarization-sensitive: this emission gets switched off when the incident polarization is rotated to 90°, *i.e.*, from *X* to *Y*, (Figure 14c) due to the sudden lack of spatial overlap between the exciting near-field and the single QD. This effect was the first demonstration of a polarization-driven single photon switch.^3^

### Lifetime Engineering

The hybrid structure shown in Figure 14a exhibited a significant Purcell factor of 24.^3^ However, since the position of the QD inside the polymer lobes is not controlled, the effective QD-nanocube distance varied randomly from sample to sample, leading to variations in the lifetime and Purcell factor of the excited state. To address this issue, a new approach was considered. Instead of mixing together the photopolymerizable formulation and the QDs, a functionalized photopolymer was used.^98^ Integrated amine groups lead to positive charges at the surface of polymerized lobes, allowing QDs to be subsequently selectively attached by immersing the hybrid system in a solution of negatively charged nanoemitters. An example of such a nanosystem is shown in Figure 14d, where about 20 QDs were integrated in the vicinity of the nanocube. The number of attached QDs is *a priori* controllable as it depends on three adjustable parameters: the size of the polymer lobes, the concentration of QDs within the solution, and the immersion time. In contrast to Figure 13c where QDs are inside the (nano)volume of the polymer, the QDs in Figure 14d are localized on the polymer surface, with the polymer thickness acting as a spacer between the QDs and the nanocube. In particular, in Figure 14d, the thickness of the polymer lobes can be considered as the average distance between the QDs and the gold nanocube surface. Since the PL lifetime is highly dependent on this distance, controlling the polymer thickness allows for the manipulation of the lifetime, as demonstrated in Figure 14e, which shows lifetime measurements on different HPNs with varying polymer thicknesses. The PL lifetime decreases with decreasing thickness, illustrating the feasibility of lifetime engineering through this approach.

**Figure 15:**
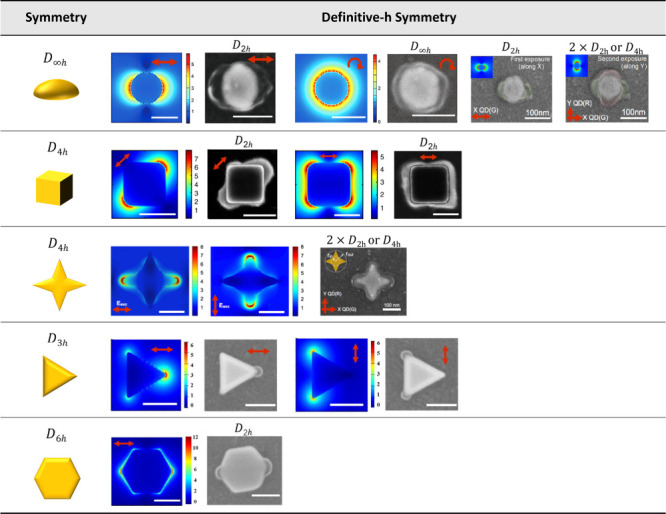
Summarizing table concerning the use of plasmon-assisted 2-photon polymerization. Left column: initial point-group symmetry of the metal nanostructures. Other columns: calculated plasmonic near-field (λ = 780 or 730 nm) used for P2PP (one or two step exposure) and resulting active medium distribution, as observed by scanning electron microscopy. The FDTD maps show the amplitude of the plasmonic near-field, except in the third line, which shows intensity maps. Two bottom lines: complementary data showing a *D*_3*h*_ symmetry Au nanotriangle whose dipolar modes can be selectively selected to structure the active medium and (last line) a *D*_6*h*_ symmetry Au multimodal nanostructure that led to a *D*_2*h*_-symmetry polymer medium containing quantum emitters. The red arrows represent the polarization used for P2PP. The white scale bars represent 100 nm.

As a summary, Figure 15 provides an illustrated overview of the different symmetry groups that have been controlled by P2PP. The left column depicts the initial point group symmetry of the metallic nanostructures. The other columns represent the resulting symmetry point groups for the active medium and the plasmonic near-field maps used for obtaining the HPNs (field amplitude). The first two rows have already been discussed in this review article with *D*_∞*h*_ and *D*_4*h*_ symmetries. The third row of the table illustrates the use of another *D*_4*h*_-symmetry gold nanostructure. In order to illustrate the flexibility of the approach, we also present here very recent achievements at the two last rows of Figure 15: the modes of a *D*_3*h*_-symmetry gold nanotriangle were used for locally structuring the active medium, in agreement with the modal description reported in ref (110). In addition, a *D*_6*h*_ symmetry gold multimodal nanostructure led to a *D*_2*h*_-symmetry polymer medium containing quantum emitters in agreement with a description of the plasmon eigenmodes by a simple group theory approach.^109^

### Concluding Remark on Approach 9

It should be stressed that this approach presents an important asset: the intrinsic plasmonic modes are used for the integration of nanoemitters, enabling a “self-positioning” of the nano-emitters. In particular, the spatial symmetry of the active medium of the hybrid nanosource of light can be designed almost on demand by selecting the plasmonic modes used for driving the nanochemistry. The specificity of this approach could be used for developing advanced chiral nanosources of light. Chirality refers to objects whose geometry and symmetry are such that the mirror image cannot coincide with the object.^113^ Chirality can be used in many domains of applications including sensing,^114^ enantiomer selectivity,^115^ and polarization design.^116^ Within the context of nanophotonics, nanoscale chirality has been explored and exploited over the past decade. Many research works were reported on plasmonic chiral nanostructures,^117,118^ but producing chiral hybrid quantum emitters is still challenging. The nanoscale polymerization approach is potentially promising for addressing this challenge. Particularly, two new avenues could be opened:

i) Non-chiral plasmonic nanostructures illuminated with circular polarization can present a chiral near-field resulting from complex mode interference.^119^ This chiral near-field can be used for locally structuring the environment to artificially make the plasmonic nanoparticles chiral.^120,121^ This approach could be used for polymer nanopositioning, via plasmonic photopolymerization, to attach nano-emitters having a chiral spatial distribution, making the resulting HPN chiral.
ii) Chiral plasmonic nanostructures can present complex near-field distribution,^122^ that can be exploited for integrating nano-emitters. Through this integration and associated coupling, the strong chirality and associated angular momentum of the plasmonic structure could be transferred to the emitted photons.

## Comparison between the Nine Different Approaches

**Figure 16:**
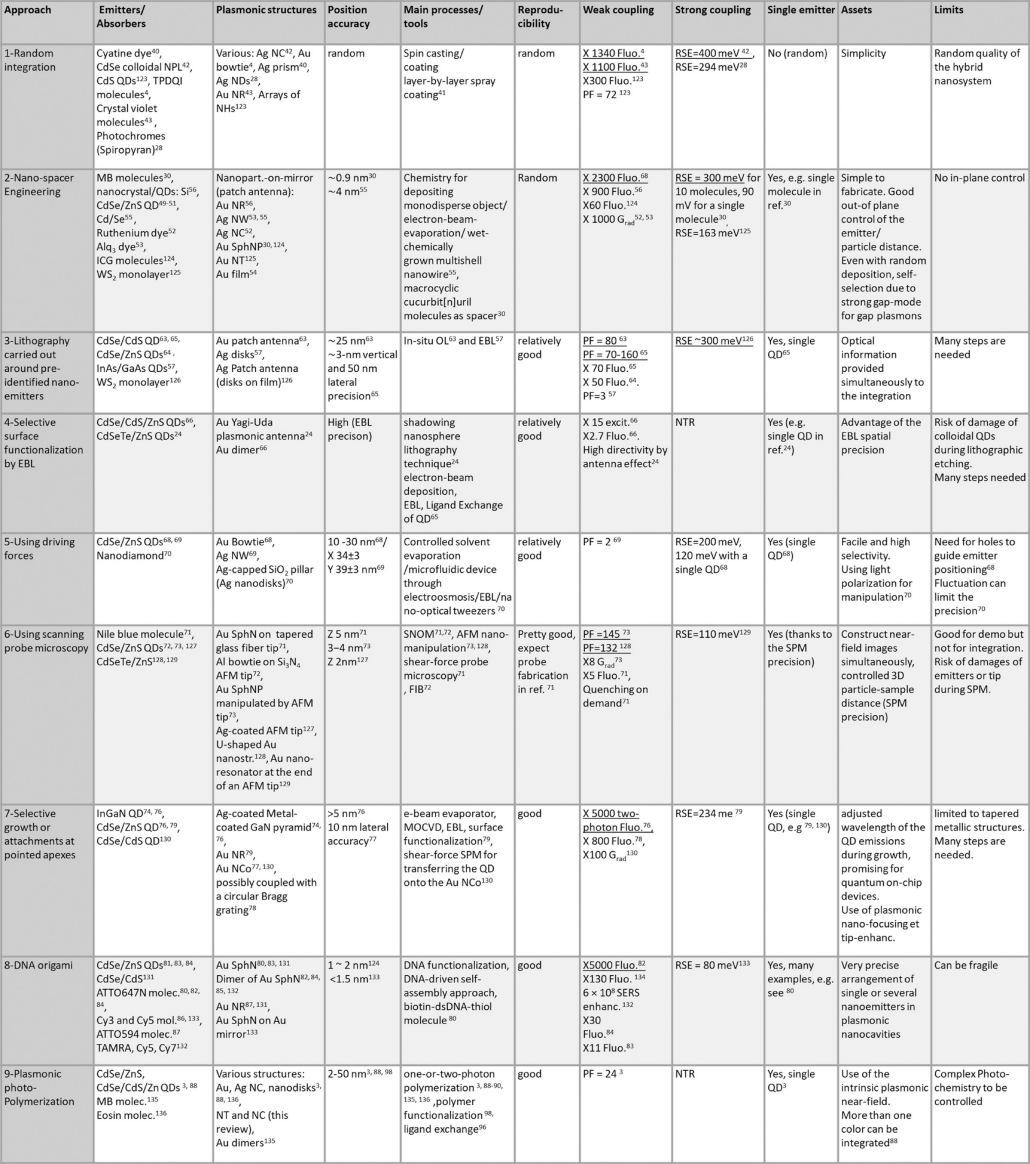
*Summary of the nine different approaches (and associated features and stated achievements) used for controlling the spatial localization on nanoemitters/absorbers in hybrid plasmonic nanosystems. Examples of the corresponding references are given. In order to complete the review, references*(123−136), *not cited in the manuscript, have been added*. The highest values reported for fluorescence intensity enhancement, Purcell factor, and Rabi splitting energy are highlighted with underline. *Acronyms used*: *AFM: Atomic Force Miscroscopy; Alq*_3_*: tris(8-hydroxyquinoline) aluminum; Enhanc.: enhancement; FIB: Focused Ion Beam; Fluo.: Fluorescence intensity; EBL: ebeam lithography; Enhanc.: enhancement. MB: methylene blue; MOCVD: Metal–Organic Chemical Vapor Deposition; Molec.: Molecule; Nanostr: nanostructure; Nanopart.: nanoparticle; NC: nano cube; NCo: nano cone; ND: nano disk; NH: nano hole; NR: nanorod; NPL nanoplatelets; NT: nano triangle; NTR: nothing to report; OL: optical lithography; PF: Purcell Factor; polymeriz.: polymerization; QD: quantum dot; RSE: Rabi splitting energy; SphNP: spherical nanoparticle*, SPM: scanning probe microscopy; TAMRA: Carboxytetramethylrhodamine; TPDDI: N,N0-bis(2,6-diisopropylphenyl)-1,6,11,16-tetra-[4(1,1,3,3-tetramethylbutyl)phenoxy]quaterrylene-3,4:13,14-bis(dicarboximide).

The nine methods are very different from each other, and each has its own strengths and limitations, making a direct comparison difficult. Each approach offers unique features and performance, which have been summarized in a table (Figure 16) that quantifies the main features and performances of the approaches. It should be noted that the table contains mainly realizations involving photoluminescence. Nevertheless, in some reported work on strong coupling, only the extinction/absorption/diffusion spectrum has been measured (*e.g.*, refs (28, 30)), whereas this regime should potentially also modify the photoluminescence properties. We have retained the nine different types of approach defined in the section “Different Approaches Developed to Address the Issue of How to Control the Positioning of the Nanoemitter/Absorber”. Ten characteristics were selected: types of nanoemitters/absorbers, types of metallic nanostructures, positioning accuracy, main processes and tools used, reproducibility, weak coupling (Purcell factor, PL enhancement, etc.), strong coupling (Rabi energy splitting), possibility of integrating a single nanoemitter, advantages, and limitations. To complete the review, new references have been added to the table.^123−136^

The reader can navigate through this table to understand the contributions of each approach to specific features or examine individual approaches for information about their potentials and limitations. More specifically, the reader can focus on a particular feature by looking at a specific column: reading the corresponding rows gives an idea of what different approaches can contribute to that feature. Alternatively, the reader can focus on a specific approach (a specific row) and scan the different columns for information about the characteristics and potential of the method.

Approach 1, which involves random integration, stands out as the simplest method, with notable results for both weak and strong coupling. To our knowledge, the highest reported Rabbi splitting energy (RSE = 400 meV^42^) and two of the best fluorescence enhancement factors (1340,^4^ 1100^43^) were obtained using this approach. However, approach 1 lacks precise control over the integration of nanoemitters, unlike approaches based on reproducible techniques such as approaches 3, 4, 7, 9.

Although control of the spacer thickness is not obvious, approach 2 is also simple to implement because, like approach 1, the active medium is often deposited randomly. Unlike approach 3, which also led to plasmonic gaps, it does not require an optical or electron-beam lithography technique. Compared with the other approaches, approach 2 takes advantage of the strong localized field of the gap mode, which has led to one of the highest values of Rabbi splitting energy (300 meV^30^). However, approach 2 does not guarantee control of the in-plane distribution of the active medium inside the gap, whereas approach 3 can, in principle, achieve this control (RSE = 300 meV was also achieved^126^), as can approaches 4 and 6, which take advantage of the extreme spatial precision of electron beam microscopy and scanning probe microscopy. Approach 4 involves defining specific areas for chemical attachment of quantum dots using local electron irradiation of the resist, followed by selective attachment of emitters. While offering precise control over the localization of the emitter, it can suffer from limitations in terms of scalability due to the complexity of the process.

Approaches 5 and 6, that use driving forces and scanning microscopy respectively, led to the highest Purcell factor value (PF = 145^73^) and one of the highest achieved RSE values (200 meV^68^). They offer elegant solutions with high accuracy but are highly dependent on probe quality and force fluctuation. Compared with the other methods, approach 7, based on selective growth at the apexes, is promising in terms of compatibility with microelectronics and mass production, at least as far as ref (74) is concerned. Its reliability is illustrated by the highest values obtained for fluorescence enhancement factors (at 2 photons) of 5000.^76^

Almost all of the approaches have demonstrated the ability to position single emitter in a relatively controlled manner, which is promising for the adaptation of single photon emitter on nanophotonic devices. Despite its limitations (in particular, the fragility of the organic matrix), approach 8 seems to be the most appropriate for the highly controlled integration of single molecule. The number of molecules can even be chosen and imposed. This high potential may explain why this method has led to the highest fluorescence enhancement factor (5000) reported to date in the literature.^82^

Finally, approach 9, which is the focus of our attention and which can be generalized to other chemical processes, is the only one that makes the best use of the intrinsic modes and the LDOS of the plasmonic nanostructure. The symmetry of the active medium can thus be controlled as can the sensitivity of the nanosystem to the polarization of light. However, this approach requires a high level of control of the involved photochemistry and has not yet demonstrated the ability to amplify fluorescence intensity, despite the demonstration of a Purcell factor of 24.^3^

In summary, each positioning approach offers unique advantages and challenges, addressing diverse applications in nanophotonics and quantum optics. The summary table and comparison provided are intended to help readers access and compare the different approaches to make suitable choices for potential hybrid plasmonic light absorber and nanosource research projects.

## Conclusion and Perspectives

Over the past 20 years, numerous methods were successfully developed for controlling the spatial localization of nanoemitters and nanoabsorbers in the close vicinity of plasmonic metal nanostructures.

Hybrid plasmonic nanosystems based on weak and strong coupling constitute a very promising family of light nanosources and nanoabsorbers. However, there are remaining challenges that could lead to breakthroughs in hybrid plasmonics based on weak and strong coupling. Three of these are briefly discussed below.

### Orientation of the Dipoles

To control weak and strong coupling, it is essential to control the orientation of the emitter or the absorber dipole. This dipole can either be the transition dipole moment,^137^ denoted as **μ** in the article, driving the transition from one state to another, through the absorption and excitation selection rules that the emitters depend on, or the optical transition dipole moment responsible for the luminescence,^138^ resulting in an emission dipole. While spherical core–shell semiconductor quantum dots possess a spherically degenerated excitation transition dipole that is isotropic in three directions (no clear defined preferential direction), they clearly present oriented emission dipoles.^139^ On the other hand, organic molecules can present defined transition dipoles for both absorption and emission.^140^ Orienting these dipoles properly relative to the plasmonic electric field in a controlled way constitutes an important challenge. Recently, using approach 8, it was demonstrated that small single molecules such as organic fluorophores can be incorporated into predefined positions of a DNA origami with controlled orientation by adjusting their linkage conditions.^141^ Some other possible avenues could rely on the use of either plasmon-based optical forces^142^ or permanent dipoles in nanocrystals^143^ that could be sensitive to DC applied fields.

### Chiral Nanosources of Light

As pointed out in the section “Focus on Approach 9: Control of the Spatial Symmetry of the Active Medium by Plasmonic Photopolymerization”, developing chiral nanosources of light is still a challenge. In addition to the already mentioned routes relying on approach 9, other methods could be used. For example, wet-chemical ligand functionalization was used to form plasmonic molecules in dispersion or induced chirality due to chemical molecules.^144^ This elegant approach could also be used for structuring the chiral spatial distribution of functionalized nanoemitters in the vicinity of the chiral plasmonic nanostructure during its formation.

As far as potential applications for chiral nanosources are concerned, ultimately a colorimetric detector of nanoscale chirality could be envisaged. Morphologically chiral colloidal plasmonic nanoantennas were produced by chirality transfer from a chiral surfactant during growth, *e.g.*, refs (145 and 146). By extension, the adsorption and subsequent excitation of chiral molecules on such an object could generate a given chiral field mode, which would translate into a specific color in the emission of QDs associated with the mode. In particular, a specific colorimetric response from a chiral HPN could be obtained depending on the chirality of a molecule close to the surface of the HPN. For example, with chiral HPNs dispersed in a medium to be analyzed, one could have the following code: left chirality = blue response, right chirality = red response, and racemic = white response. This approach could lead to many potential applications for synthesis in chemistry, biology, and even medicine.

### Three Colors HPN

In Figure 12, we commented on the integration of two kinds of QDs, leading to polarization-sensitive 2-color HPN presenting a *D*_4*h*_ symmetry that was obtained from gold nanodisks. This allowed one to control color emission and energy transfer from donor-QDs (green) and acceptor-QDs (red).^88^

**Figure 17:**
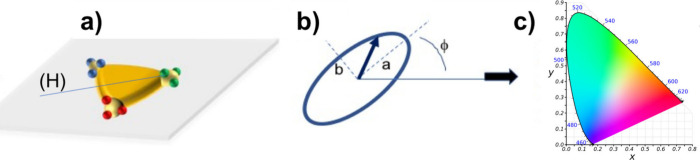
A promising perspective: integration of 3 colors on the same *D*_3*h*_-symmetry nanostructure. (a) Schematic representation of the structure. (b) Elliptical polarization used for exciting the nanostructure, characterized by (ϕ, *a*:*b*). (c) International 1931 CIE color space diagram.

Using a *D*_3*h*_-symmetry plasmonic nanostructure, it would be challenging, using one of the nine approaches or a combination of some of them, to integrate three kinds of QDs at different sites and directions, leading to a 3-color nano pixel (Figure 17a) of specific symmetry that can be commanded with an elliptical polarization illustrated in Figure 17b. Let us consider the 3-color hybrid system illustrated in Figure 17a, illuminated by the incident wavelength λ_inc_. The weight of the far-field-emitted color, *i* (*i* ∈ 1–3), *W*_*i*_, is proportional to γ_*exci*_*abs*_*i*_*QY*_*i*_*transf*_*i*_, where γ_exci_ is the local excitation rate related to the local near-field at λ_inc_. It depends on (ϕ, *a*:*b*) and includes possible plasmonic enhancement factors. For example, in Figure 17a, a linear polarization along the (*H*) axis is expected to select mainly green QDs while a circular polarization will excite all of the QDs. abs_i_ is the absorption coefficient at λ_inc_. QY_i_ is the quantum yield, and transf_*i*_ is related to any possible energy transfers between QDs of color i and the other QDs.^88^

In principle, any state of polarization (ϕ,*a*:*b*) could lead to a specific effective color within the international 1931 CIE color space diagram shown in Figure 17c, resulting from Σ*W*_i_, the far-field mixing between different colors. The (ϕ,*a*:*b*)/*W*_i_ correlation will have to be fully quantified, leading to a database which will be valuable for envisaged applications. For example, a silica-coated 3-color HPN could be used as an integrated tunable nanosource for local spectroscopy. Reciprocally, another application would be the counterpart of the nanosource aspect. A self-assembly of HPN RGB pixels (of the same orientation) would constitute a colorimetric detector of linear, circular, and elliptical polarization in the CIE space.
